# Inhibitors of cytochrome *c* biogenesis pathways

**DOI:** 10.1128/mbio.00273-26

**Published:** 2026-04-20

**Authors:** Deanna L. Mendez, Pema L. Childs, Amidala J. Martinie, Jonathan Q. Huynh, Andy F. Zhu, Samuel R. McKee, George S. Ghabrial, Christina L. Stallings, Robert G. Kranz

**Affiliations:** 1 Department of Biology, Washington University in St Louis 7548, St. Louis, Missouri, USA; 2 Department of Molecular Microbiology, Center for Women's Infectious Disease Research, Washington University School of Medicine 12275 , Saint Louis, Missouri, USA; The University of Utah, Salt Lake City, Utah, USA

**Keywords:** heme, heme trafficking, cytochrome *c*, heme attachment, cytochrome c assembly

## Abstract

The unique composition of bacterial electron transport chains (ETCs), distinct from the human mitochondrial ETC, presents opportunities for selective antibacterial targeting. Recently, inhibitors of bacterial ETCs have shown promise as potential antimicrobials, particularly against mycobacteria. Notably, bacterial cytochrome c (cyt *c*) biogenesis systems—Systems I and II—are absent in mammalian mitochondria, which utilize System III. This divergence suggests that cyt *c* biosynthesis pathways may serve as novel antibiotic targets. In this study, we screened a library of 1,760 FDA-approved compounds for inhibitors of *in vivo* bacterial cyt *c* biogenesis. We identified seven compounds that inhibit either System I and/or System II pathways. Among these, artemisinin and its analogs (ARTs) were found to directly inhibit *in vitro* cyt *c* biogenesis using purified CcsBA (System II) and human HCCS (System III). ARTs disrupt heme at the active sites of cyt *c* synthases, indicating their potential as direct inhibitors. However, therapeutic use of ARTs would likely require co-administration with inhibitors targeting alternative bacterial respiratory chains that do not involve cyt *c*. Additionally, three members of the 8-hydroxyquinoline (8-HQ) class—clioquinol, chloroxine, and broxyquinoline—inhibited *in vivo* cyt *c* biogenesis at low micromolar concentrations, below those needed to suppress bacterial growth. Results suggest that 8-HQs act through mechanisms beyond simple metal chelation (e.g., iron, magnesium), likely targeting multiple bacterial processes. Consistent with this hypothesis, 8-HQs did not directly inhibit *in vitro* cyt *c* synthases.

**IMPORTANCE:**

This manuscript establishes bacterial cytochrome c biogenesis as a viable and previously underexplored antibacterial target that is fundamentally distinct from human mitochondrial pathways. Through systematic screening of 1,760 FDA-approved compounds, the study identifies two chemically and mechanistically distinct inhibitor classes—artemisinins and 8-hydroxyquinolines—that disrupt cytochrome c maturation via Systems I and II. The work moves beyond phenotypic growth inhibition by directly linking compound activity to heme degradation using complementary *in vivo* assays and *in vitro* experiments with purified cytochrome c synthases. Demonstrating that artemisinins directly target heme within these enzymes provides mechanistic insight with broad relevance to bacterial bioenergetics and drug-heme interactions. Importantly, the manuscript highlights the metabolic flexibility of bacterial respiratory chains and shows that inhibition of cytochrome c biogenesis alone is insufficient for robust killing. Together, these findings argue that cytochrome c biogenesis inhibitors will be effective in combination therapies and advance understanding of bacterial respiration.

## INTRODUCTION

The urgent need for new antibiotics to combat bacterial pathogens has been well documented over the past few decades, primarily due to the growing prevalence of antimicrobial resistance (e.g., references 1–4). Since the discovery of penicillin nearly a century ago, numerous strategies for antibiotic discovery have emerged (e.g., references 5–9). Natural product screening from soil and other environments has yielded many important antimicrobial agents (reviewed in references 10–13). In addition, targeted approaches focusing on specific bacterial processes, such as transcription and translation, have proven successful (1, 14–17), along with broader searches for compounds that impede bacterial growth (18, 19).

More recently, components of bacterial energy metabolism—particularly electron transport chains (ETCs) and ATP synthesis—have emerged as promising targets. For example, Q203, an inhibitor of cytochrome *bc*_1_, and aurachin D, which targets cytochrome bd, have been shown to suppress the growth of various mycobacterial species (20–24). Many bacteria possess ETCs that are structurally and functionally distinct from the mitochondrial ETC of their hosts (25–33), providing opportunities for selective inhibition. Most bacteria possess c-type cytochromes as components of ETCs. Some ETCs in bacteria are like the classic aerobic ETC in mitochondria, but other ETCs (and their specific c-type cytochromes) are used in anaerobic metabolisms. For c-type cytochromes, the heme group is covalently attached at a conserved CXXCH motif, a process that requires a cytochrome c synthase for biogenesis.

**Fig 1:**
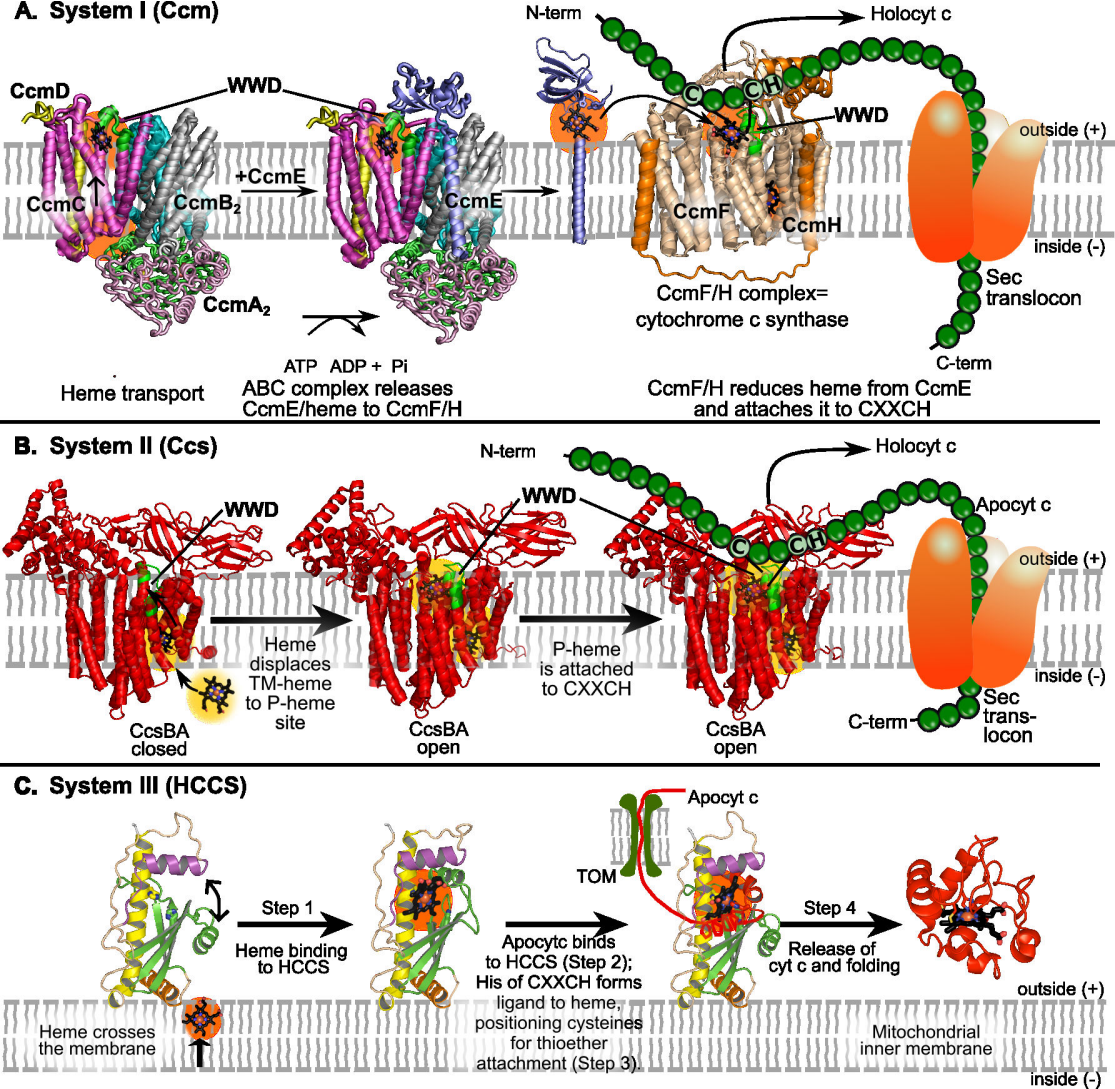
Three cyt *c* biogenesis pathways: Systems I, II, and III. (**A**) System I is composed of eight Ccm proteins; CcmABCD exports heme to CcmE, which then delivers heme to the CcmF/H cyt *c* synthase. System I is in many gram-negative bacteria. (**B**) System II is a bifunctional CcsBA heme exporter and cyt *c* synthase. It is present in many gram-positive bacteria, cyanobacteria, mycobacteria, bacteroides, and some gram-negative bacteria. (**C**) System III is composed of HCCS, present in the mitochondrial intermembrane space of eukaryotes. Taken from (34).

We have proposed that inhibiting cytochrome c (cyt *c*) biogenesis could disrupt multiple ETCs that rely on c-type cytochromes (34). Bacterial cyt *c* biogenesis occurs via pathways known as Systems I and II, which are mechanistically distinct from the simpler mitochondrial pathway (System III) (Fig. 1A through C) (reviewed in references 35–41). Selectively targeting Systems I and II could therefore enable bacteria-specific inhibition. These pathways operate in the periplasm, akin to peptidoglycan synthesis, making them accessible to drugs (Fig. 1A and B). In both systems, heme is exported from the cytoplasm to the periplasm, where it is covalently attached via thioether bonds to apocytochrome c at a conserved CXXCH motif before folding occurs. Recent structural advances, including high-resolution Cryo-EM and X-ray crystal structures of most proteins involved in Systems I and II, have laid the foundation for rational inhibitor design (Fig. 1A and B) (34, 42–46).

In this study, we use recombinant Systems I and II to identify inhibitors of cyt *c* biogenesis. We screened the Pharmakon-1760 compound library—comprising 1,760 structurally diverse, biologically active drugs approved in the United States, Europe, or Asia (http://www.msdiscovery.com/pharmakon.html)—to identify inhibitors of cyt *c* maturation. Two compound classes, artemisinins (ARTs) and 8-hydroxyquinolines (8-HQs), were found to inhibit cyt *c* synthesis at low micromolar concentrations. We evaluate their inhibitory effects *in vivo* using recombinantly expressed Systems I, II, and III in *Escherichia coli*, and *in vitro* with purified Systems II and III. We employ mitochondrial System III since, ultimately, it may be necessary to find compounds that preferentially inhibit the bacterial pathways. Additionally, we assess their impact on bacterial growth and discuss their potential mechanisms of action and implications for future development of ETC-targeting antibiotics.

## RESULTS

### *In vivo* screening of recombinant *E. coli* strains for inhibitors of cytochrome c biogenesis Systems I and II

The Pharmakon-1760 compound library, described in the Materials and Methods section, was used to screen for inhibitors of cyt *c* biogenesis Systems I and II. The screen was conducted using two independent sets of 96-well microtiter plates, each containing a total of 1,760 compounds at a final concentration of 7.5 μM. One set was used to identify inhibitors of System I, and the other for System II.

As illustrated in Fig. S1, *E. coli* strains lacking the endogenous *ccmA-H* operon (System I) were complemented with plasmids expressing either the *E. coli* System I genes (*ccmA-H*) or the *Helicobacter hepaticus* System II (*ccsBA*) genes (47, 48). These recombinant strains were optimized for cyt *c* biosynthesis by induction with IPTG and co-transformed with a separate plasmid carrying a gene encoding the cyt *c*_4_ reporter protein, also under inducible control.

Cultures were grown overnight, back-diluted, and induced with IPTG, and 200 μL aliquots were transferred into wells containing individual compounds. Arabinose was added to induce expression of cyt *c*_4_ in each well, enabling cyt *c* biogenesis to proceed overnight. Inhibitors affecting transcription, translation, or the maturation pathways were expected to yield decreased cyt *c*_4_ levels and were thus identified as positive hits.

**Fig 2:**
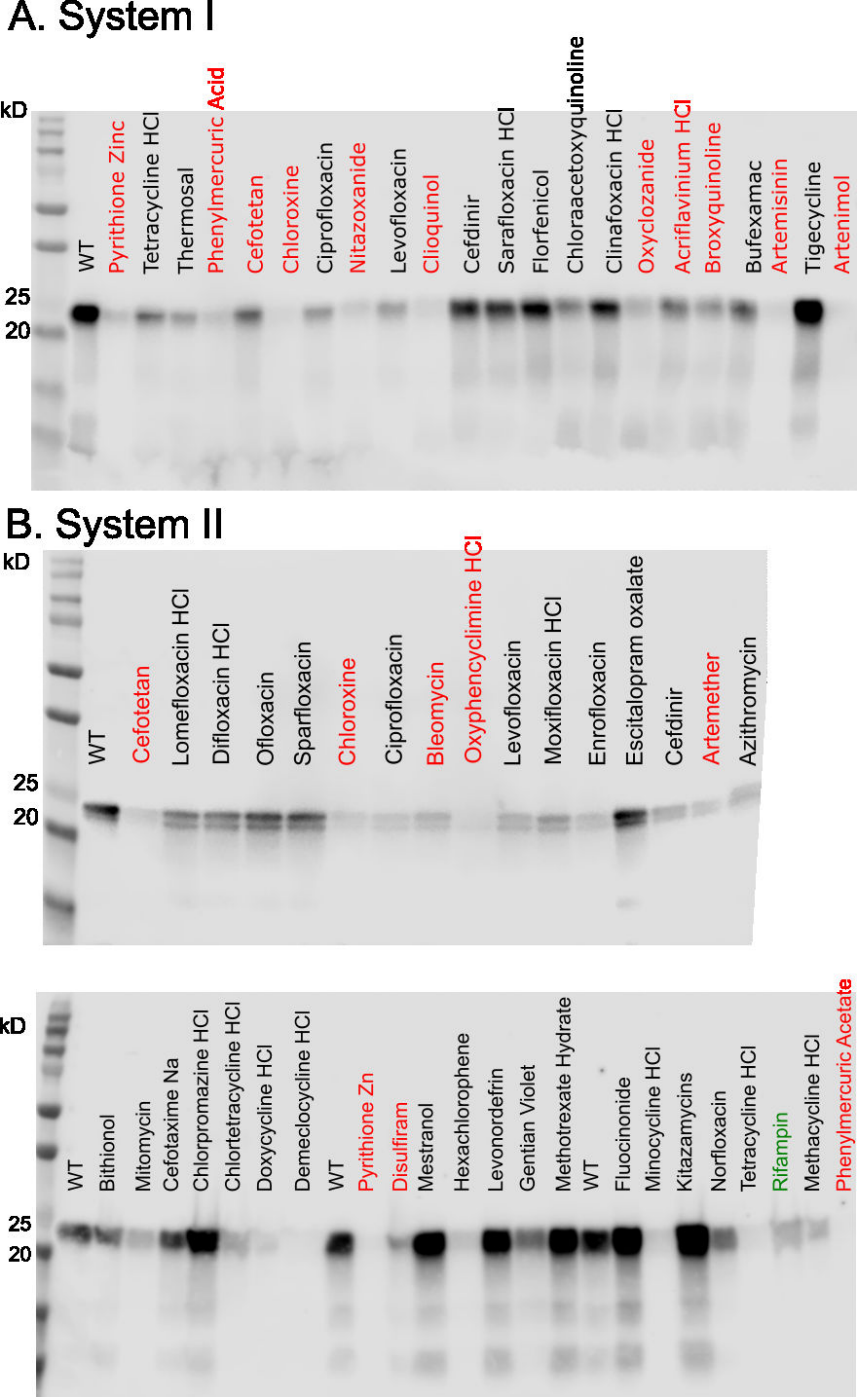
Response of a subset of Pharmakon-1760 compounds in cytochrome *c*_4_ assays. Heme stains of SDS-PAGE gels are shown, with the heme-stained band detecting heme attachment mediated by the indicated cyt *c* biogenesis system. Candidate inhibitors are shown in red. (**A**) Recombinant *E. coli* Δ*ccm* with System I producing cyt *c*_4_ in the presence of the listed drugs. (**B**) Recombinant *E. coli* Δ*ccm* with System II producing cyt *c*_4_. Rifampicin (green font) is a transcriptional inhibitor in the Pharmakon-1760 library that inhibits protein expression. kD refers to the molecular weights, with 20 kDa and 25 kDa markers shown.

**Fig 3:**
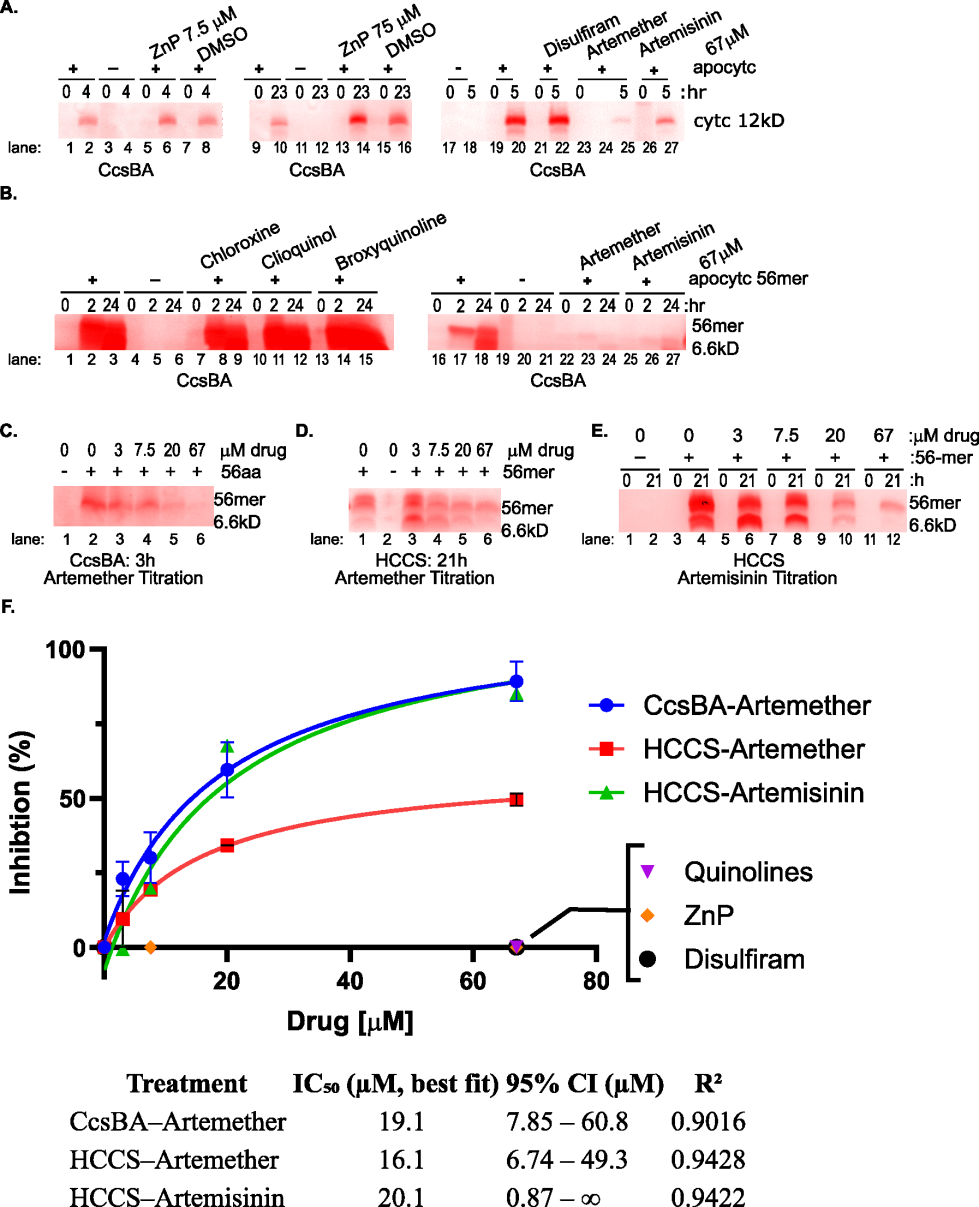
Inhibition of cyt *c* maturation by artemether and artemisinin *in vitro*. Either the purified System II CcsBA or human System III HCCS was used in cyt *c* biogenesis assays as described in Materials and Methods. (**A and B**) *In vitro* biosynthesis of cyt *c* by CcsBA was carried out in an anaerobic chamber. The heme stain visualizes heme-containing proteins (cyt *c* or 56-mer). Reactions performed in the presence of artemether and artemisinin (panel A, lanes 25 and 27, and panel B, lanes 23, 24, 26, 27) show inhibited cyt *c* maturation. In contrast, no inhibition was observed for pyrithione zinc (ZnP) (panel A, lanes 6, 14), disulfiram (panel A, lane 22), or the quinolines: chloroxine (panel B, lanes 8, 9), clioquinol (panel B, lanes 11, 12), or broxyquinoline (panel B, lanes 14, 15). (**C–E**) Titrations of artemether and artemisinin with CcsBA (**C**) or HCCS (**D and E**) showed a concentration-dependent inhibition. (**F**) A plot of the data presented in panels **A–E** ImageJ was used to measure the heme stain density. The signal in a given lane was normalized by the reaction signal in the absence of drug on a particular gel. Artemether-CcsBA (*n* = 3); HCCS-artemether (*n* = 2); HCCS-artemisinin (*n* = 1); CcsBA-ZnP (*n* = 1), CcsBA-disulfiram (*n* = 3); CcsBA-quinolines (*n* = 3). Error bars are the standard error of the mean (SEM). Determination of IC_50_ values for *in vitro* inhibition of cytochrome c biogenesis by artemether and artemisinin. Dose-response curves were fit using a three-parameter nonlinear regression model in GraphPad Prism.

Following incubation, cultures were centrifuged, and cell pellets were lysed using B-PER bacterial lysis buffer. Samples were analyzed by SDS-PAGE, followed by heme-specific staining, which selectively detects heme covalently attached to cyt *c*_4_ via Systems I or II (Fig. S1). All *c*-type cytochromes contain heme that is covalently attached to the cytochrome *c* polypeptide. This attachment is catalyzed specifically by the cyt *c* synthase of each biogenesis system (Fig. 1). Because the covalently bound heme remains associated with the polypeptide after denaturing SDS-PAGE, it can be detected using a heme-specific stain. Accordingly, the bands shown in Fig. 2 and 3 represent a sensitive readout of cyt *c* production and cyt *c* synthase activity.

As described below, Fig. 2 shows heme-stained bands (following SDS-PAGE) from whole *E. coli* cells expressing either System I or System II; thus, these experiments serve as *in vivo* assays for cyt *c* biogenesis. To determine whether the compounds act directly on cyt *c* synthases, we also performed *in vitro* activity assays using purified synthases. In these assays, covalent heme attachment was again assessed by heme staining after SDS-PAGE (Fig. 3).

Compounds that resulted in reduced cyt *c*_4_ levels were selected for further validation. B-PER lysates were reanalyzed by SDS-PAGE and heme staining, and the intensity of the cyt *c*_4_ heme signal was quantified (Fig. 2; Table 1). Compounds with known antibacterial targets, such as tetracyclines (translation inhibitors) and rifampicin (transcription inhibitor), were excluded from further consideration.

**TABLE 1:** Compounds from the Pharmakon-1760 collection with indicated cyt *c* biogenesis inhibition^*a*^

Gel panel	Drug name	System I % heme signal/WT	System II % heme signal/WT
i	Acriflavinium hydrochloride	41	60*
ii	Artemether	47*	15
iii	Artenimol	6	36*
iv	Artemisinin	6	43*
v	Bleomycin	59*	31
vi	Broxyquinoline	28	92*
vii	Chloroxine	4	22
viii	Clioquinol	7	61*
ix	Cefotetan	41	15
x	Disulfiram	73*	35
xi	Nitazoxanide	7	85*
xii	Oxyclozanide	18	111*
xiii	Oxyphencyclimine	99*	11
xiv	Phenylmercuric acid/acetate	10	2
xv	Pyrithione zinc	14	12

^*a*^ An asterisk indicates that the heme signal was calculated based on the original Pharmakon-1760 gel; all others are calculated based on those in Fig. 2. Shading indicates compounds with no previously characterized targets in bacteria were prioritized for follow-up studies.

From the screen, 15 compounds that produced a reduction in heme signal to 42% or less of the control were selected for further analysis (Table 1; compound structures shown in Table S1). Of these, seven compounds with no previously characterized targets in bacteria were prioritized for follow-up studies (shaded in gray in Table 1).

### Drug effects on *in vitro* cytochrome c biogenesis via purified System II (CcsBA) and System III (human HCCS)

To evaluate the specific effects of selected compounds on cyt *c* biogenesis, we investigated their impact on *in vitro* cyt *c* synthesis using purified System II (CcsBA) and System III (human HCCS) enzymes (49). These assays employed either full-length apocytochrome c or a previously characterized 56-amino-acid peptide containing the CXXCH motif as the heme acceptor (Fig. 3) (49).

Among the tested compounds, pyrithione zinc (ZnP), disulfiram, and the three 8-HQs—chloroxine, clioquinol, and broxyquinoline—did not inhibit cyt *c* synthase activity, even at concentrations exceeding 50 μM (Fig. 3A and B). In contrast, both artemisinin (ART) derivatives, artemether and artemisinin, exhibited significant inhibitory effects, with approximate IC₅₀ values of ~20 µM (Fig. 3C through F).

To further explore the mechanism of inhibition by ARTs, we analyzed the spectroscopic signatures of heme during the *in vitro* reactions catalyzed by CcsBA and HCCS (Fig. S2). Both synthases harbor b-type heme at their active sites, which initially yields characteristic absorption maxima near 560 nm (Fig. S2Aii, Bii, Ci, and Di). Formation of c-type heme, involving thioether linkages, produces a distinct absorption maximum in the 550–555 nm range, distinguishable from the 560 nm peak of b-type heme (Fig. S2Ai, Bi, Cii, and Dii) (46, 48–51).

In the absence of ARTs, the expected spectral transitions and heme levels were consistent with normal enzymatic activity—that is, a shift from b-heme to c-heme characteristics as the reaction proceeded. However, in the presence of ARTs, a dose-dependent degradation of heme was observed for both CcsBA and HCCS reactions (Fig. S2C and SD). Consistent with this, heme staining of cyt *c* and the 56 mer peptide following SDS-PAGE revealed minimal heme attachment in ART-treated samples (Fig. 3). This aligns with the marked reduction of the 550 nm peak (compare Fig. S2Ai through vi and vii).

Additionally, the Soret band (~420 nm), which reflects both b-type and c-type heme species, showed decreased intensity with increasing artemether concentrations (Fig. S2Avi, Bvi, and Cvi), indicating an overall reduction in heme content. These findings suggest that ARTs directly interact with the heme bound within the active site of the cyt *c* synthases.

This observation parallels the known mechanism of ARTs in *Plasmodium* species, where they inhibit hemozoin formation by forming covalent adducts with heme (e.g., references 52–66). Based on these results, we propose that ARTs are activated by heme within CcsBA and HCCS, leading to heme adduct formation, heme degradation, and ultimately the inactivation of cyt *c* synthase activity. We do not observe ARTs-dependent protein degradation (e.g., Fig. S3B, Coomassie-stained gel 1, compare *CcsA:His, CcsB* bands in lane 5 to lanes 10 and 12; gel 2, compare lane 5 to lanes 9 and 11; gel 3, compare lane 3 to lanes 5 and 6).

### Analysis of growth inhibition of *E. coli* and determination of IC₅₀ values *in vivo* for selected drugs targeting cyt *c* biogenesis

To investigate the potential of selected compounds that inhibit cyt *c* biogenesis *in vivo*, we focused on two representatives from each drug class, ARTS (artemisinin and artemether) and 8-HQs (chloroxine and clioquinol). We assessed their effects in recombinant *E. coli* strains engineered to express cytochrome *c* maturation Systems I, II, or III (Fig. S1).

To further evaluate the specific impact on cyt *c* biogenesis of each drug, we selected concentrations below levels that inhibited growth (confirmed via OD_600_ measurements; Fig. S4). Thus, OD_600_ measurements indicate that protein synthesis and stability are not impacted by the drugs. Because most apocyt c proteins are degraded if heme is not attached (39), assaying for heme attached to the cyt *c* protein using heme stains of SDS-PAGE is a direct measure of cyt *c* synthase activity.

**Fig 4:**
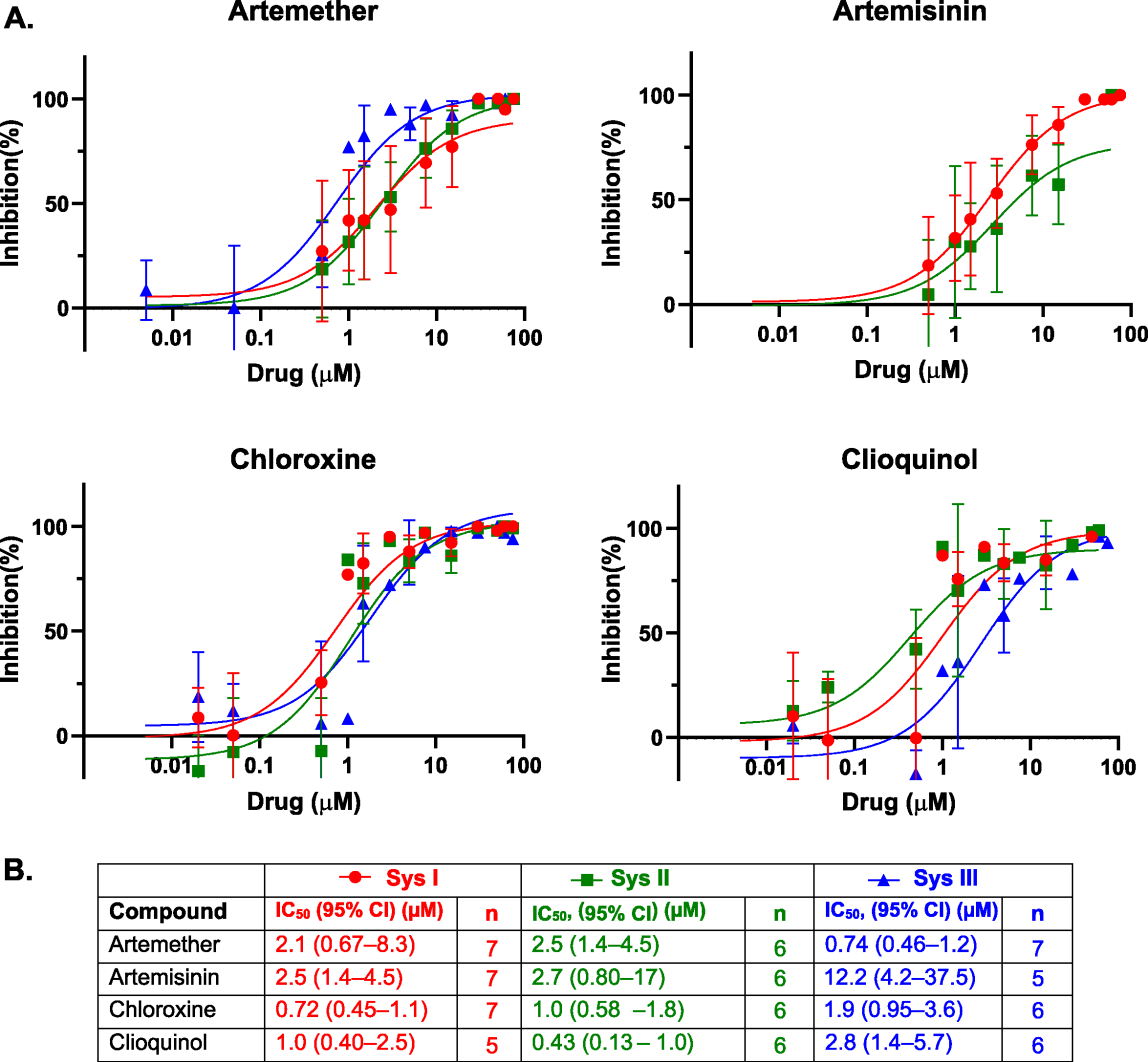
Dose-response analysis of inhibitor activity against cytochrome *c* biogenesis Systems I–III *in vivo* in *E. coli* Δ*ccm*. Heme stain assays for cyt *c* with the indicated concentrations of drugs were performed as shown in Fig. S5. (**A**) Nonlinear regression curves showing the response of Systems I (red circles), II (green squares), and III (blue triangles) to increasing concentrations of candidate inhibitors. Data points represent the mean ± SD from independent replicates, with *n* shown in the table. Each curve was fitted with a three-parameter logistic model to determine half-maximal inhibitory concentrations (IC_50_). (**B**) Summary of the fitted IC_50_ values (with 95% confidence intervals).

To determine IC_50_ values for *in vivo* cyt *c* biogenesis, *E. coli* strains lacking the eight endogenous System I *ccm* genes were transformed with plasmid-borne maturation Systems I, II, or III. Note that *E. coli* Δccm strains are incapable of producing c-type cytochromes unless complemented with a System but remain viable due to alternative terminal oxidases (cyt *bd* and cyt *bo*). *E. coli* does not require *c*-type cytochromes for aerobic growth. Cultures were induced following the Pharmakon screening protocol (Fig. S1) with the volume of 5 mL, with compounds added after IPTG induction and 30 minutes prior to arabinose addition. Cyt *c*_4_ levels were assessed by SDS-PAGE followed by heme staining (Fig. S5). A summary of IC_50_ values is presented in Fig. 4.

Artemether and artemisinin inhibited cyt *c* biogenesis via Systems I and II with IC_50_ values ranging from 2.1 to 2.7 μM. Notably, artemether also inhibited System III (human HCCS) with an IC_50_ of approximately 0.7 μM. In contrast, artemisinin showed inhibition for System III about 15-fold weaker at 12.2 μM. This differential activity may result from limited membrane permeability of artemisinin compared to artemether. Unlike Systems I and II, which function in the periplasm (Fig. 1), the human HCCS catalyzes heme attachment in the cytoplasm. For Systems I and II, cyt *c*_4_ is equipped with a signal sequence for periplasmic targeting, whereas human cyt *c* lacks such a sequence and remains cytoplasmic.

Our results demonstrate that ARTs inhibit cyt *c* biogenesis *in vivo* at low micromolar concentrations—consistent with their *in vitro* inhibitory effects on both CcsBA and HCCS (Fig. 3).

Chloroxine and clioquinol inhibited bacterial Systems I and II with IC₅₀ values in the submicromolar range (0.43 μM–1.0 μM), well below concentrations that impair *E. coli* growth. In System III, clioquinol exhibited a higher IC_50_ of 2.8 μM—approximately threefold higher than for the bacterial systems. These findings suggest that while both ARTs and 8-HQs interfere with cyt *c* maturation, their potency and mechanisms of action may differ depending on the maturation system and subcellular localization of the target enzyme.

### Inhibition of bacterial growth as a proxy to identify potential targets and antibiotics

**Fig 5:**
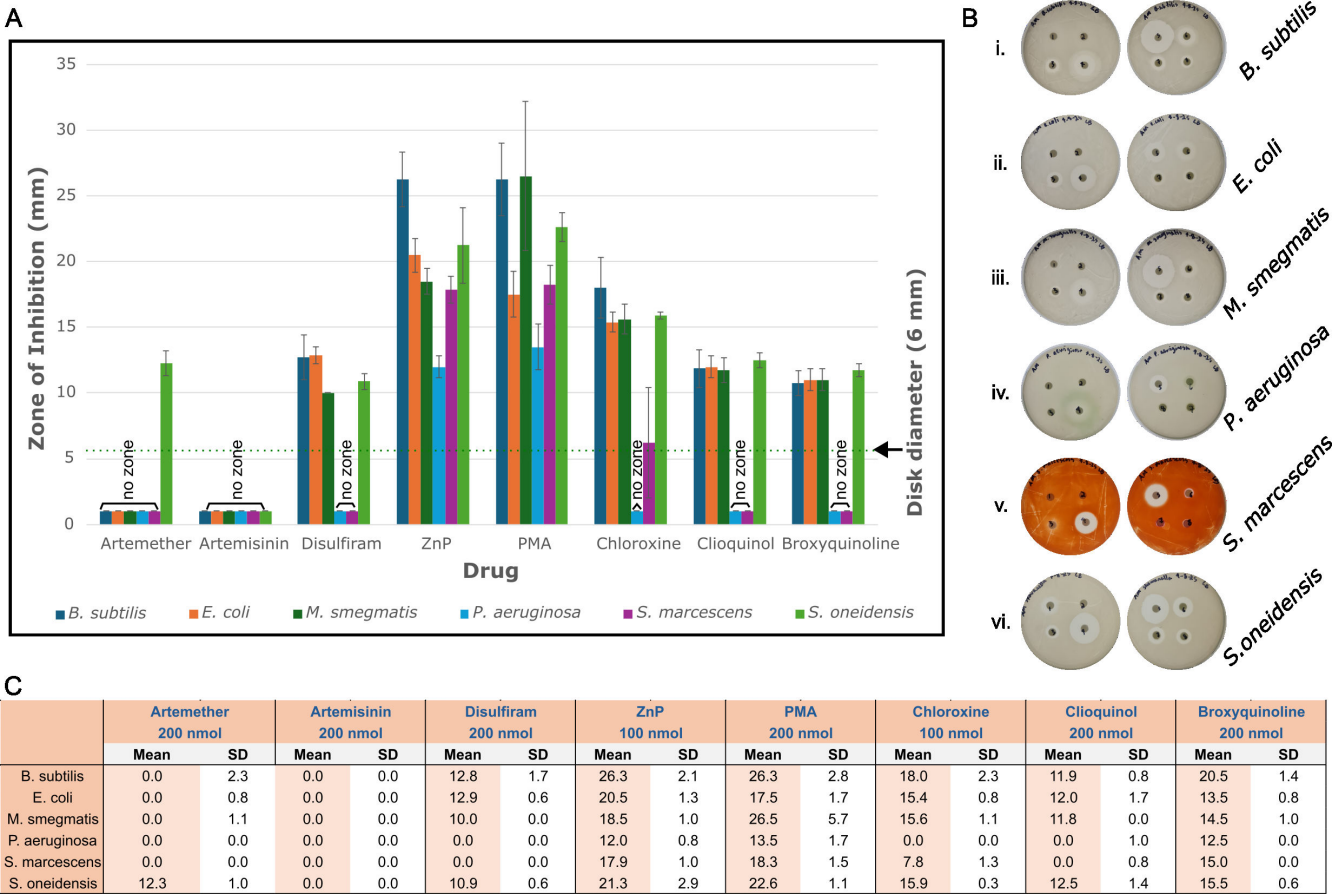
Zones of inhibition produced by eight candidate cytochrome *c* biogenesis inhibitors on six bacterial strains with distinct respiratory systems. (**A**) Bar graph shows the mean inhibition diameter (mm) from *n* = 4 independent biological replicates. (**B**) Representative agar plates showing the primary data used to measure zones of inhibition for the indicated bacterial strains, as described in Materials and Methods. (**C**) Summary of the drug concentration, the mean zone of inhibition, and standard deviations. ZnP, pyrithione zinc; PMA, phenyl mercuric acetate.

Eight compounds were selected for further evaluation of antibacterial activity, including two artemisinin derivatives (ARTs: artemisinin and artemether), three 8-HQs: clioquinol, chloroxine, and 8-bromoquinoline), and three additional compounds from Table 1. Antibacterial activity was assessed using disk diffusion assays to measure zones of inhibition (Fig. 5).

We tested six phylogenetically diverse bacteria, chosen for their distinct respiratory systems and health relevance: two gram-positive species—*Bacillus subtilis* and *Mycobacterium smegmatis* (both utilizing cytochrome *c* biogenesis System II)—and four gram-negative species—*Escherichia coli*, *Pseudomonas aeruginosa*, *Serratia marcescens*, and *Shewanella oneidensis* (all using System I). *Shewanella* was included due to its unusually high c-type cytochrome content, which supports aerobic, anaerobic, and nanowire ETCs (e.g., references 67–70). All six bacteria were cultured aerobically, as detailed in Materials and Methods. Their ETC composition and cytochrome usage are well-characterized (e.g., references 29, 33, 71).

Figure 5 shows representative inhibition zones for all eight compounds across the six bacterial species; full data sets are presented in Fig. S6. All compounds except the ARTs exhibited zones of inhibition in at least one bacterial species. Disulfiram, pyrithione zinc (ZnP), phenyl mercuric acetate (PMA), and all three 8-HQs demonstrated antibacterial activity, suggesting broad-spectrum or selective inhibitory effects.

Given that 8-HQs are established metal chelators (e.g., references 72–75) and that cytochrome *c* biogenesis requires iron for heme synthesis, we further investigated whether their mechanism of action is primarily via metal chelation. To assess this, we compared their activity profiles with known metal chelators, including the iron chelator dipyridyl and the general chelator EDTA (Fig. S7). Our rationale was that if the mechanism of action of the 8-HQs is as specific metal chelators, the zones should be similar relative to the known chelators among the six bacteria.

Interestingly, the antibacterial profiles of the 8-HQs did not align with those of dipyridyl or EDTA. For example, none of the 8-HQs produced inhibition zones against *P. aeruginosa* or *S. marcescens*, whereas dipyridyl yielded large zones in both species. Conversely, *M. smegmatis* showed sensitivity to all 8-HQs but was unaffected by dipyridyl. *E. coli* and *S. oneidensis* were highly sensitive to dipyridyl but not to 8-HQs. EDTA exhibited broad activity across all species.

We also determined IC₅₀ and IC₉₀ values for the inhibition of *Mycobacterium tuberculosis* (Mtb) by artemisinin, artemether, clioquinol, and chloroxine (Table 2; Fig. S8). Consistent with the *M. smegmatis* data, *M. tuberculosis* was sensitive to the 8-HQs but resistant to ARTs.

**TABLE 2:** MABA^*b*^ IC_50_ values

Compound	IC_50_ ± SD (µM)	IC_90_ ± SD (µM)
Artemether	299.034 ± 2.090	378.309 ± 2.449
Artemisinin	386.306 ± 86.230	501.350 ± 121.408
Clioquinol	9.580 ± 0.124	11.728 ± 0.196
Chloroxine	5.122 ± 0.602	6.561 ± 0.739
INH	0.284 ± 0.013	ND^*a*^

^*a*^ ND, unable to calculate INH IC_90_ due to insufficient data points.

^*b*^ MABA, microplate alamar blue assay.

These results suggest that if the 8-HQs acted solely through metal chelation, their inhibition patterns would mirror those of other chelators. Since they do not, we propose that 8-HQs may act through multiple mechanisms, including inhibition of cytochrome c biogenesis (see Discussion).

In contrast, the ARTs exhibited minimal antibacterial activity, with only artemether showing a zone of inhibition—and only against *S. oneidensis*. Two possible explanations may account for this limited activity: (i) functional redundancy in bacterial respiratory chains, where multiple terminal oxidases (e.g., c-type, bd-type, or a-type) allow respiration to continue despite partial ETC inhibition (34); or (ii) poor compound permeability, limiting ARTs’ access to intracellular or periplasmic targets.

To explore this further, we tested whether artemether affects cytochrome *c* synthesis in *S. oneidensis*. Cells were grown aerobically in increasing concentrations of artemether, and c-type cytochrome levels were analyzed via SDS-PAGE and heme staining. Even at sub-inhibitory concentrations, artemether reduced c-type cytochrome levels (Fig. S9), indicating specific inhibition of the cytochrome *c* biogenesis pathway. This also implies that artemether reaches the periplasm, where System I components such as CcmF and CcmH are localized (see Fig. 1A).

## DISCUSSION

We previously demonstrated that CcsBA requires only a CXXCH peptide as a substrate, whereas human HCCS requires a longer peptide that includes both the CXXCH motif and an upstream α-helix (49). These findings, based on *in vitro* reconstitution of bacterial CcsBA and human HCCS, align with *in vivo* data using HCCS substrates (76, 77). Additionally, we showed that short CXXCH peptides lacking the extended α-helix act as inhibitors of CcsBA activity *in vitro*, but not of HCCS (49). The proposed mechanism involves these short peptides serving as competitive substrates, thereby consuming heme that would otherwise be incorporated into full-length apocytochrome c. Whether these short peptides also inhibit CcsBA *in vivo* remains uncertain, though peptide accessibility to the periplasm likely complicates such investigations. Another unresolved question is whether these peptides cause dead-end inhibition of the synthase. Modifying the CXXCH peptides with electrophilic “warheads” could potentially enhance inhibition, but again, periplasmic access and interference from cellular thiols present significant technical hurdles.

To expand the search for inhibitors of cytochrome c biogenesis, we conducted *in vivo* screens using a library of 1,760 compounds approved for human use across a broad range of biological targets. Among the hits (Table 1), we identified two distinct classes of compounds—ARTs and 8-HQs—each represented by three active members that inhibited cytochrome c biogenesis via Systems I and/or II. We first discuss the ARTs.

Our results show that ARTs inhibit cytochrome c biogenesis *in vivo* across Systems I, II, and III, with IC₅₀ values in the low micromolar range (Fig. 4). *In vitro* assays using purified CcsBA and human HCCS confirmed direct inhibition of synthase activity, likely via interaction of ARTs with the heme cofactors of the synthases. Drawing on a substantial body of literature describing ART-heme adduct formation in malaria parasites (e.g., references 52–66), our findings support the hypothesis that similar ART-heme adducts disrupt cyt *c* synthase activity. We have not observed degradation of the cyt *c* synthase proteins themselves in our *in vitro* assays. This suggests that the major reaction of ARTs is with the heme in the cyt *c* synthases, at least early on in reactions.

While our study was ongoing, a separate report described that ARTs may chemically rescue a respiratory-deficient *Saccharomyces cerevisiae* Bcs1 mutant by reducing cyt *bc*_₁_ overaccumulation (78). This mutant accumulates inactive cyt *bc*_₁_ complexes due to impaired Bcs1 chaperone function. Notably, ARTs reduced cyt *bc*_₁_ levels in both yeast and human BCS1 mutants, suggesting a therapeutic potential for certain mitochondrial disorders. That study also reported covalent ART adducts with cytochrome c. Although we cannot exclude the possibility of ARTs targeting cytochrome c directly, our *in vitro* data point to the heme cofactors in cyt *c* synthases as the primary molecular targets.

A recent report showed that ARTs permeate both gram-positive and gram-negative bacteria and exhibit bactericidal activity against *Vibrio cholerae* but not *E. coli*, *P. aeruginosa*, or *B. subtilis* (79). Interestingly, copper was shown to sensitize bacteria to ARTs in that study. In our experiments, only *Shewanella* exhibited zones of inhibition in response to artemether. At micromolar concentrations, artemether led to decreased levels of c-type cytochromes in *Shewanella*. These findings suggest a potential for ARTs or related compounds to serve as inhibitors of bacterial Systems I or II in future antimicrobial development.

The second inhibitor class identified through our *in vivo* screens was the 8-HQs. While 8-HQs have long been known to inhibit bacterial growth, their precise mechanism of action has remained unclear (e.g., references 80, 81). Recent studies suggest that 8-HQ activity requires aerobic growth and is enhanced in the presence of zinc, with superoxide dismutase (SOD) implicated as a potential target (82). Given that 8-HQs are known metal chelators, we considered whether their antibacterial activity might result from sequestering essential metals such as iron, thereby indirectly inhibiting heme synthesis and, consequently, cytochrome c biogenesis.

To investigate this, we compared bacterial sensitivity to known iron chelators (e.g., dipyridyl) with sensitivity to 8-HQs across a diverse panel of bacterial strains (Fig. S7). No clear correlation was observed, suggesting that metal chelation alone does not account for the activity of 8-HQs. While we do not rule out metal chelation as a contributing factor, our results support a more complex mechanism involving multiple cellular targets. At varying concentrations, 8-HQs may act through a combination of iron chelation, SOD inhibition, and disruption of quinone-mediated electron transport. Importantly, our results show that 8-HQs inhibit cytochrome c biogenesis, although this is unlikely to be the sole mechanism of growth inhibition.

This conclusion is supported by our observation that *E. coli*, which lacks a functional cytochrome c biogenesis pathway and does not rely on a c-type cytochrome-based aerobic electron transport chain, remains sensitive to all tested 8-HQs. Thus, while cytochrome c biogenesis may be a relevant target, additional cellular processes are likely affected by 8-HQs.

Given the metabolic flexibility of bacteria, particularly in their respiratory chains, we propose that inhibitors of cytochrome c biogenesis alone are unlikely to exhibit broad-spectrum antibacterial activity. Many bacteria have evolved redundant and versatile electron transport chains (e.g., references 34, 83, 84), allowing them to bypass inhibition of any single component. Indeed, among the species tested, only *Shewanella*, which relies heavily on c-type cytochromes, exhibited clear susceptibility to ARTs. This highlights the challenge of targeting cytochrome c biogenesis as a standalone antimicrobial strategy.

As demonstrated in studies on mycobacteria (30, 85–87), effective targeting of bacterial respiration may require combination therapies that inhibit multiple components of the electron transport chain. The precedent set by inhibitors of cytochrome *bc*_1_ (22, 30, 86, 88) and cytochrome bd (89–91) underscores the promise of bioenergetic targets in antimicrobial development. While most bacterial electron transport chains require heme, not all rely on c-type cytochromes. Thus, inhibition of heme biosynthesis itself, as proposed in recent studies (92), may offer a broader strategy. However, some pathogens can scavenge heme from their host, which may limit the efficacy of such approaches.

In conclusion, the diversity and adaptability of bacterial respiratory pathways necessitate multi-target strategies for the development of effective antibiotics. Inhibiting cytochrome c biogenesis could be one component of a broader approach targeting multiple respiratory enzymes and pathways.

## MATERIALS AND METHODS

### Pharmakon-1760 screen

The Pharmakon-1760 library (1.5 nmol of each drug) was tested for the ability to inhibit cyt *c* biogenesis by System I or System II in a volume of 200 μL (final drug concentration of 7.5 μM) (MicroSource Discovery Systems, Inc.).

*E. coli* with System I or System II was grown overnight to saturation in a rocking incubator. That culture was back-diluted into Luria Bertani broth in a 1:5 ratio and grown for 3 hours at 37°C and 200 rpm. The expression of System I or System II was induced with 0.1 mM IPTG and continued shaking for 1 hour. Two hundred microliters of *E. coli* was aliquoted into the plate wells. Note columns 1 and 12 do not contain any drugs. We used column 1 for a positive control (System I, II, and cyt *c*) and column 12 as a negative control (no cyt *c* plasmid in *E. coli*). The drug was allowed to interact with the *E. coli* for 30 minutes. Subsequently, cyt *c* expression was induced with 0.2% arabinose. The plate was incubated overnight with shaking. *E. coli* was harvested by centrifugation the following day. The supernatant was emptied from the plate and placed at −80°C.

To extract cyt *c* and test for the efficacy of the test drug, the *E. coli* was thawed and resuspended in 40 µL of B-PER Complete Bacterial Protein Extraction Reagent (Thermo Fisher Scientific)**.**

### In-gel heme stain

Samples were run on 4-20% or 15% gels purchased from Bio-Rad. An in-gel heme stain showed the presence of matured cyt *c* (band at 24 kDa) in the following way: The gel was rinsed with deionized water and then fixed with Coomassie Destain (40% water, 50% methanol, and 10% acetic acid) for 5 minutes. Forty-five milligrams of 3,3′,5,5′-tetramethylbenzidine (TMBZ) was dissolved in 22.5 mL of methanol with rocking. It was then diluted to 75 mL with 0.2 M acetate buffer pH 3. After fixing the gel, it was incubated in the TMBZ solution for 15 minutes. Finally, 450 µL of hydrogen peroxide was added to the gel to develop the heme-stainable bands for 10 minutes. The gel was then imaged with a Fuji camera. Hits were identified that had less cytochrome c expression compared to the positive control.

### *In vitro* experiments

Experiments were performed as previously described (49). In brief, samples were put into an anaerobic chamber (N_2_ [95%] and H_2_ [5%] in a Coy anaerobic airlock chamber) and allowed to equilibrate for 30 minutes. CcsBA (or HCCS) was combined with apo cyt *c* or a 56-mer peptide that contains the heme attachment site. Samples were imaged with a UV spectrophotometer, and 10 μL was collected and placed in 10 μL loading buffer to run as T = 0 on an SDS-PAGE gel. The samples were then incubated at 37°C for 4 to 24 hours. Longer time allowed for the signal of cyt *c* to dominate the heme signature as CcsBA-heme signal is lost over time. The reaction was then imaged by UV-vis spectrometry. Ten microliters was collected and placed in gel loading buffer.

Gel samples were run on an SDS-page gel, rinsed, and imaged using the in-gel heme stain method with TMBZ.

### IC_50_ determination by heme stain of cyt *c* biogenesis pathways

In the follow-up experiments to determine the IC_50_ of the candidate drugs, we followed the same protocol that we used for the plates. We back-diluted a saturated overnight culture into the number of test tubes that we required for the experiment at a ratio of 1:5 with a 5 mL volume. We allowed the culture to enter the log phase by growing it for 3 hours. Then we induced the expression of Systems I, II, or III with 0.1 mM IPTG. Unique to System III, the rotation speed was reduced after induction to 120 rpm from 200 rpm. Then the *E. coli* culture was added to the respective drugs in test tubes for 30 minutes, at which time 0.2% arabinose was added to the culture to induce the expression of cyt *c*. Cultures were grown overnight and then harvested by centrifugation.

Samples were stored at −80°C until processing. For lysis, samples were thawed at room temperature in the presence of B-PER Complete reagent for 15 minutes. The pellet was then resuspended by vortexing and incubated for an additional 15 minutes at room temperature. Insoluble material was removed by centrifugation at 16,000 × *g* for 10 minutes; this step was repeated three times. The final supernatant was collected and analyzed by SDS–PAGE.

### Heme stain analysis by transfer

For the IC_50_ determination, the protein from SDS-PAGE was transferred to a PVDF membrane. SuperSignal West Femto Maximum Sensitivity Substrate (Thermo Fisher Scientific) was used to detect the presence of heme. The chemiluminescence was detected using a LI-COR Odyssey (LI-COR).

### Densitometry

Band densitometry was detected using the built-in software on the LI-COR software. A user-defined section of the gel was used as background. The band density was determined and exported. ImageJ was used in some instances to determine band densitometry, for example, *Shewanella*, and for in-gel heme stain quantitation from *in vitro* experiments (93).

### Zones of inhibition

To obtain information about a range of bacteria that respond to our drugs of interest, we employed a zone of inhibition experiment. In this experiment, 50 μL of an overnight culture (37°C for *E. coli-C43* Δccm, *M. smegmatis-*general laboratory strain (gls), *P. aeruginosa-*gls, 30°C for *S. marcescens-*ATCC 43862, *S. oneidensis-*gls, *B. subtilis-*gls) was plated onto a petri culture dish (size, 10 cm) of Luria Bertani. A filter disk was then placed on the plate, and 10 μL of the respective drugs was added to the disk. Plates were incubated at their respective temperatures until a zone was observed or a lack thereof. These methods have been widely used, and the low standard deviations of the measured zones (e.g., Fig. 5C) support their validity. As noted in the text, some bacterial species were selected because their cytochrome c compositions are well-characterized, even though they are not clinical pathogens.

### IC_50_ determination by microplate alamar blue assay (MABA) in Mtb

MABAs were performed with Mtb as previously described (88, 94, 95). Briefly, a culture of wild-type Mtb Erdman strain was initiated by inoculating 200 µL of frozen stock into 40 mL of Difco Middlebrook 7H9 liquid medium supplemented with 5 g/L BSA, 2 g/L dextrose, 0.003 g/L catalase, 0.85 g/L sodium chloride, 0.5% glycerol, and 0.05% Tween 80. The culture was grown in a 1 L roller bottle and incubated at 37°C while rolling for 5 days until it reached logarithmic growth phase at an OD_600_ of approximately 0.6. It was then diluted in 7H9-T media (7H9 media with Tween 80) to an OD_600_ of 0.0016. From this dilution, 100 µL was added to each well of a 96-well plate containing 100 µL of serially diluted test compounds prepared in 7H9-T. The final DMSO concentration was normalized to 1% across all wells. To minimize edge effects due to evaporation, the outermost wells were filled with 200 µL of 7H9-T to serve as a moat.

The microplates were incubated in a humidified incubator at 37°C and 5% CO_2_ for 7 days. After incubation, 32.5 µL of a solution consisting of 0.6 mM resazurin and 20% Tween 80 in an 8:5 ratio was added to each well. Plates were returned to the incubator for an additional 24 hours. Fluorescence was then measured using a BioTek Synergy H1 Microplate Reader (excitation: 530 nm; emission: 590 nm).

Relative fluorescence units (RFU) for each well were converted to percent inhibition using the following formula:


%inhibition=[1−(w−n)/(p−n)]×100%

where *w* is RFU of the well of interest, *p* is the average RFU of wells containing Mtb only (positive control), and *n* is the average RFU of wells containing media only (negative control).

Compound inhibition curves were generated using GraphPad Prism, and a non-linear regression model was manually fitted using Microsoft Excel Solver to estimate the IC_50_ and IC_90_.

### *In vivo* assay of cytochrome response in *Shewanella oneidensis*

Overnight cultures of *S. oneidensis* were 10-fold diluted into 5 mL of LB with eight different concentrations of artemether (5 mM, 500 μM, 50 μM, 5 μM, 500 nM, 50 nM, 5 nM, 0 M). The cultures were grown at 200 rpm at 30°C for 5 hours and harvested. The bacteria were frozen overnight at −80°C. The protein was extracted with 200 μL of B-PER complete. The spectra were measured, and 10 μL were run on an SDS-PAGE gel from each sample. The protein was transferred to PVDF (Bio-Rad) and imaged with SuperSignal West Femto reagent (Thermo Fisher Scientific). The same blot was then imaged with Sypro Ruby (Bio-Rad) to see total protein levels. Data were collected from three independent experiments. The percent inhibition was plotted assuming complete cytochrome c inhibition at 5 mM. The data were fitted with a three-parameter logistic model to determine IC₅₀. The 95% confidence interval for the IC_50_ is reported in Fig. S9C.

## SUPPLEMENTAL MATERIAL

Supplemental material mbio.00273-26-s0001.pdf Table S1; Figures S1-S9.

ASM does not own the copyrights to Supplemental Material that may be linked to, or accessed through, an article. The authors have granted ASM a non-exclusive, world-wide license to publish the Supplemental Material files. Please contact the corresponding author directly for reuse.

## References

[1] Miethke M, Pieroni M, Weber T, Brönstrup M, Hammann P, Halby L, Arimondo PB, Glaser P, Aigle B, Bode HB, et al.. 2021. Towards the sustainable discovery and development of new antibiotics. Nat Rev Chem 5:726–749. doi:10.1038/s41570-021-00313-1 PMC8374425 34426795

[2] Smith WPJ, Wucher BR, Nadell CD, Foster KR. 2023. Bacterial defences: mechanisms, evolution and antimicrobial resistance. Nat Rev Microbiol 21:519–534. doi:10.1038/s41579-023-00877-3 37095190

[3] Uddin TM, Chakraborty AJ, Khusro A, Zidan BRM, Mitra S, Emran TB, Dhama K, Ripon MKH, Gajdács M, Sahibzada MUK, Hossain MJ, Koirala N. 2021. Antibiotic resistance in microbes: history, mechanisms, therapeutic strategies and future prospects. J Infect Public Health 14:1750–1766. doi:10.1016/j.jiph.2021.10.020 34756812

[4] Waglechner N, Culp EJ, Wright GD. 2021. Ancient antibiotics, ancient resistance. EcoSal Plus 9:eESP-0027-2020. doi:10.1128/ecosalplus.ESP-0027-2020 PMC11163840 33734062

[5] Gootz TD. 1990. Discovery and development of new antimicrobial agents. Clin Microbiol Rev 3:13–31. doi:10.1128/CMR.3.1.13 2404566 PMC358138

[6] Hutchings MI, Truman AW, Wilkinson B. 2019. Antibiotics: past, present and future. Curr Opin Microbiol 51:72–80. doi:10.1016/j.mib.2019.10.008 31733401

[7] Ramírez-Rendon D, Passari AK, Ruiz-Villafán B, Rodríguez-Sanoja R, Sánchez S, Demain AL. 2022. Impact of novel microbial secondary metabolites on the pharma industry. Appl Microbiol Biotechnol 106:1855–1878. doi:10.1007/s00253-022-11821-5 35188588 PMC8860141

[8] Roemer T, Davies J, Giaever G, Nislow C. 2011. Bugs, drugs and chemical genomics. Nat Chem Biol 8:46–56. doi:10.1038/nchembio.744 22173359

[9] Shim H. 2023. Three innovations of next-generation antibiotics: evolvability, specificity, and non-immunogenicity. Antibiotics (Basel) 12:204. doi:10.3390/antibiotics12020204 36830114 PMC9952447

[10] Lewis K, Lee RE, Brötz-Oesterhelt H, Hiller S, Rodnina MV, Schneider T, Weingarth M, Wohlgemuth I. 2024. Sophisticated natural products as antibiotics. Nature 632:39–49. doi:10.1038/s41586-024-07530-w 39085542 PMC11573432

[11] Prusov EV. 2016. Natural product-based antibiotics: synthesis and SAR-studies. Curr Pharm Des 22:1730–1755. doi:10.2174/1381612822666160115130633 26769331

[12] Townsley L, Shank EA. 2017. Natural-product antibiotics: cues for modulating bacterial biofilm formation. Trends Microbiol 25:1016–1026. doi:10.1016/j.tim.2017.06.003 28688575 PMC5701842

[13] Walker AS, Clardy J. 2024. Primed for discovery. Biochemistry 63:2705–2713. doi:10.1021/acs.biochem.4c00464 39497571 PMC11542185

[14] Hartmann G, Behr W, Beissner KA, Honikel K, Sippel A. 1968. Antibiotics as inhibitors of nucleic acid and protein synthesis. Angew Chem Int Ed Engl 7:693–701. doi:10.1002/anie.196806931 4177609

[15] Ho JM, Bakkalbasi E, Söll D, Miller CA. 2018. Drugging tRNA aminoacylation. RNA Biol 15:667–677. doi:10.1080/15476286.2018.1429879 29345185 PMC6103670

[16] Prezioso SM, Brown NE, Goldberg JB. 2017. Elfamycins: inhibitors of elongation factor-Tu. Mol Microbiol 106:22–34. doi:10.1111/mmi.13750 28710887 PMC5701666

[17] Vázquez-Laslop N, Mankin AS. 2018. Context-specific action of ribosomal antibiotics. Annu Rev Microbiol 72:185–207. doi:10.1146/annurev-micro-090817-062329 29906204 PMC8742604

[18] Mullis MM, Rambo IM, Baker BJ, Reese BK. 2019. Diversity, ecology, and prevalence of antimicrobials in nature. Front Microbiol 10:2518. doi:10.3389/fmicb.2019.02518 31803148 PMC6869823

[19] Ortmayr K, de la Cruz Moreno R, Zampieri M. 2022. Expanding the search for small-molecule antibacterials by multidimensional profiling. Nat Chem Biol 18:584–595. doi:10.1038/s41589-022-01040-4 35606559

[20] de Jager VR, Dawson R, van Niekerk C, Hutchings J, Kim J, Vanker N, van der Merwe L, Choi J, Nam K, Diacon AH. 2020. Telacebec (Q203), a new antituberculosis agent. N Engl J Med 382:1280–1281. doi:10.1056/NEJMc1913327 32212527

[21] Nguyen TQ, Hanh BTB, Jeon S, Heo BE, Park Y, Choudhary A, Moon C, Jang J. 2022. Synergistic effect of Q203 combined with PBTZ169 against Mycobacterium tuberculosis. Antimicrob Agents Chemother 66:e0044822. doi:10.1128/aac.00448-22 36321819 PMC9765072

[22] Pethe K, Bifani P, Jang J, Kang S, Park S, Ahn S, Jiricek J, Jung J, Jeon HK, Cechetto J, et al.. 2013. Discovery of Q203, a potent clinical candidate for the treatment of tuberculosis. Nat Med 19:1157–1160. doi:10.1038/nm.3262 23913123

[23] Yanofsky DJ, Di Trani JM, Król S, Abdelaziz R, Bueler SA, Imming P, Brzezinski P, Rubinstein JL. 2021. Structure of mycobacterial CIII2CIV2 respiratory supercomplex bound to the tuberculosis drug candidate telacebec (Q203). elife 10. doi:10.7554/eLife.71959 PMC8523172 34590581

[24] Zhou S, Wang W, Zhou X, Zhang Y, Lai Y, Tang Y, Xu J, Li D, Lin J, Yang X, Ran T, Chen H, Guddat LW, Wang Q, Gao Y, Rao Z, Gong H. 2021. Structure of Mycobacterium tuberculosis cytochrome bcc in complex with Q203 and TB47, two anti-TB drug candidates. elife 10:e69418. doi:10.7554/eLife.69418 34819223 PMC8616580

[25] Dunn AK. 2023. Alternative oxidase in bacteria. Biochim Biophys Acta Bioenerg 1864:148929. doi:10.1016/j.bbabio.2022.148929 36265564

[26] Esposti MD. 2020. On the evolution of cytochrome oxidases consuming oxygen. Biochim Biophys Acta Bioenerg 1861:148304. doi:10.1016/j.bbabio.2020.148304 32890468

[27] Franza T, Gaudu P. 2022. Quinones: more than electron shuttles. Res Microbiol 173:103953. doi:10.1016/j.resmic.2022.103953 35470045

[28] Hein S, Simon J. 2019. Bacterial nitrous oxide respiration: electron transport chains and copper transfer reactions. Adv Microb Physiol 75:137–175. doi:10.1016/bs.ampbs.2019.07.001 31655736

[29] Kranz RG, Beckett CS, Goldman BS. 2002. Genomic analyses of bacterial respiratory and cytochrome c assembly systems: Bordetella as a model for the system II cytochrome c biogenesis pathway. Res Microbiol 153:1–6. doi:10.1016/s0923-2508(01)01278-5 11881892

[30] Lee B.S, Singh S, Pethe K. 2023. Inhibiting respiration as a novel antibiotic strategy. Curr Opin Microbiol 74:102327. doi:10.1016/j.mib.2023.102327 37235914

[31] Martín-Rodríguez AJ. 2023. Respiration-induced biofilm formation as a driver for bacterial niche colonization. Trends Microbiol 31:120–134. doi:10.1016/j.tim.2022.08.007 36075785

[32] Price EE, Román-Rodríguez F, Boyd JM. 2021. Bacterial approaches to sensing and responding to respiration and respiration metabolites. Mol Microbiol 116:1009–1021. doi:10.1111/mmi.14795 34387370 PMC8638366

[33] Richardson DJ. 2000. Bacterial respiration: a flexible process for a changing environment. Microbiology (Reading) 146 (Pt 3):551–571. doi:10.1099/00221287-146-3-551 10746759

[34] Kranz RG, Sutherland MC. 2025. Mechanisms and control of heme transport and incorporation into cytochrome c Annu Rev Microbiol 79:23–43. doi:10.1146/annurev-micro-050624-031631 40460019

[35] Allen JWA. 2011. Cytochrome c biogenesis in mitochondria--systems III and V. FEBS J 278:4198–4216. doi:10.1111/j.1742-4658.2011.08231.x 21736702

[36] Babbitt SE, Sutherland MC, San Francisco B, Mendez DL, Kranz RG. 2015. Mitochondrial cytochrome c biogenesis: no longer an enigma. Trends Biochem Sci 40:446–455. doi:10.1016/j.tibs.2015.05.006 26073510 PMC4509832

[37] Hamel P, Corvest V, Giegé P, Bonnard G. 2009. Biochemical requirements for the maturation of mitochondrial c-type cytochromes. Biochim Biophys Acta 1793:125–138. doi:10.1016/j.bbamcr.2008.06.017 18655808

[38] Kranz R, Lill R, Goldman B, Bonnard G, Merchant S. 1998. Molecular mechanisms of cytochrome c biogenesis: three distinct systems. Mol Microbiol 29:383–396. doi:10.1046/j.1365-2958.1998.00869.x 9720859

[39] Kranz RG, Richard-Fogal C, Taylor J-S, Frawley ER. 2009. Cytochrome c biogenesis: mechanisms for covalent modifications and trafficking of heme and for heme-iron redox control. Microbiol Mol Biol Rev 73:510–528, doi:10.1128/MMBR.00001-09 19721088 PMC2738134

[40] Sanders C, Turkarslan S, Lee D-W, Daldal F. 2010. Cytochrome c biogenesis: the Ccm system. Trends Microbiol 18:266–274. doi:10.1016/j.tim.2010.03.006 20382024 PMC2916975

[41] Simon J, Hederstedt L. 2011. Composition and function of cytochrome c biogenesis system II. FEBS J 278:4179–4188. doi:10.1111/j.1742-4658.2011.08374.x 21955752

[42] Brausemann A, Zhang L, Ilcu L, Einsle O. 2021. Architecture of the membrane-bound cytochrome c heme lyase CcmF. Nat Chem Biol 17:800–805. doi:10.1038/s41589-021-00793-8 33958791 PMC7611092

[43] Huynh JQ, Lowder EP, Kranz RG. 2023. Structural basis of membrane machines that traffick and attach heme to cytochromes. J Biol Chem 299:105332. doi:10.1016/j.jbc.2023.105332 37827288 PMC10663686

[44] Ilcu L, Denkhaus L, Brausemann A, Zhang L, Einsle O. 2023. Architecture of the heme-translocating CcmABCD/E complex required for cytochrome c maturation. Nat Commun 14:5190. doi:10.1038/s41467-023-40881-y 37626034 PMC10457321

[45] Li J, Zheng W, Gu M, Han L, Luo Y, Yu K, Sun M, Zong Y, Ma X, Liu B, Lowder EP, Mendez DL, Kranz RG, Zhang K, Zhu J. 2022. Structures of the CcmABCD heme release complex at multiple states. Nat Commun 13:6422. doi:10.1038/s41467-022-34136-5 36307425 PMC9616876

[46] Mendez DL, Lowder EP, Tillman DE, Sutherland MC, Collier AL, Rau MJ, Fitzpatrick JAJ, Kranz RG. 2022. Cryo-EM of CcsBA reveals the basis for cytochrome c biogenesis and heme transport. Nat Chem Biol 18:101–108. doi:10.1038/s41589-021-00935-y 34931065 PMC8712405

[47] Feissner RE, Richard-Fogal CL, Frawley ER, Loughman JA, Earley KW, Kranz RG. 2006. Recombinant cytochromes c biogenesis systems I and II and analysis of haem delivery pathways in Escherichia coli. Mol Microbiol 60:563–577. doi:10.1111/j.1365-2958.2006.05132.x 16629661

[48] Frawley ER, Kranz RG. 2009. CcsBA is a cytochrome c synthetase that also functions in heme transport. Proc Natl Acad Sci USA 106:10201–10206. doi:10.1073/pnas.0903132106 19509336 PMC2700922

[49] Sutherland MC, Mendez DL, Babbitt SE, Tillman DE, Melnikov O, Tran NL, Prizant NT, Collier AL, Kranz RG. 2021. In vitro reconstitution reveals major differences between human and bacterial cytochrome c synthases. eLife 10:e64891. doi:10.7554/eLife.64891 33973521 PMC8112865

[50] Babbitt SE, San Francisco B, Bretsnyder EC, Kranz RG. 2014. Conserved residues of the human mitochondrial holocytochrome c synthase mediate interactions with heme. Biochemistry 53:5261–5271. doi:10.1021/bi500704p 25054239 PMC4139152

[51] San Francisco B, Bretsnyder EC, Kranz RG. 2013. Human mitochondrial holocytochrome c synthase’s heme binding, maturation determinants, and complex formation with cytochrome c. Proc Natl Acad Sci USA 110:E788–97. doi:10.1073/pnas.1213897109 23150584 PMC3587199

[52] Azargoshasb H, Lee H-J, Sullivan DJ, Rimer JD, Vekilov PG. 2025. The hematin-dihydroartemisinin adduct mobilizes a potent mechanism to suppress β-hematin crystallization. J Biol Chem 301:110310. doi:10.1016/j.jbc.2025.110310 40449598 PMC12271862

[53] Borstnik K, Paik I-H, Posner GH. 2002. Malaria: new chemotherapeutic peroxide drugs. Mini Rev Med Chem 2:573–583. doi:10.2174/1389557023405620 12370042

[54] Francis SE, Sullivan DJJ, Goldberg DE. 1997. Hemoglobin metabolism in the malaria parasite Plasmodium falciparum. Annu Rev Microbiol 51:97–123. doi:10.1146/annurev.micro.51.1.97 9343345

[55] Hong YL, Yang YZ, Meshnick SR. 1994. The interaction of artemisinin with malarial hemozoin. Mol Biochem Parasitol 63:121–128. doi:10.1016/0166-6851(94)90014-0 8183310

[56] Kannan R, Sahal D, Chauhan VS. 2002. Heme-artemisinin adducts are crucial mediators of the ability of artemisinin to inhibit heme polymerization. Chem Biol 9:321–332. doi:10.1016/s1074-5521(02)00117-5 11927257

[57] Liu X, Cao J, Huang G, Zhao Q, Shen J. 2019. Biological activities of artemisinin derivatives beyond malaria. Curr Top Med Chem 19:205–222. doi:10.2174/1568026619666190122144217 30674260

[58] Ma W, Balta VA, West R, Newlin KN, Miljanić OŠ, Sullivan DJ, Vekilov PG, Rimer JD. 2021. A second mechanism employed by artemisinins to suppress Plasmodium falciparum hinges on inhibition of hematin crystallization. J Biol Chem 296:100123. doi:10.1074/jbc.RA120.016115 33239360 PMC7949059

[59] Meshnick SR. 1994. The mode of action of antimalarial endoperoxides. Trans R Soc Trop Med Hyg 88 Suppl 1:S31–2. doi:10.1016/0035-9203(94)90468-5 8053021

[60] Pandey AV, Tekwani BL, Singh RL, Chauhan VS. 1999. Artemisinin, an endoperoxide antimalarial, disrupts the hemoglobin catabolism and heme detoxification systems in malarial parasite. J Biol Chem 274:19383–19388. doi:10.1074/jbc.274.27.19383 10383451

[61] Ribbiso KA, Heller LE, Taye A, Julian E, Willems AV, Roepe PD. 2021. Artemisinin-based drugs target the Plasmodium falciparum heme detoxification pathway. Antimicrob Agents Chemother 65:e02137-20. doi:10.1128/AAC.02137-20 33495226 PMC8097475

[62] Robert A, Benoit-Vical F, Claparols C, Meunier B. 2005. The antimalarial drug artemisinin alkylates heme in infected mice. Proc Natl Acad Sci USA 102:13676–13680. doi:10.1073/pnas.0500972102 16155128 PMC1224611

[63] Robert A, Coppel Y, Meunier B. 2002. Alkylation of heme by the antimalarial drug artemisinin. Chem Commun 5:414–415. doi:10.1039/b110817b 12120518

[64] Rodriguez M, Claparols C, Robert A, Meunier B. 2002. Alkylation of microperoxidase-11 by the antimalarial drug artemisinin. Chembiochem 3:1147–1149. doi:10.1002/1439-7633(20021104)3:11<1147::AID-CBIC1147>3.0.CO;2-8 12404644

[65] Selmeczi K, Robert A, Claparols C, Meunier B. 2004. Alkylation of human hemoglobin A0 by the antimalarial drug artemisinin. FEBS Lett 556:245–248. doi:10.1016/s0014-5793(03)01448-0 14706857

[66] Yang J, He Y, Li Y, Zhang X, Wong Y-K, Shen S, Zhong T, Zhang J, Liu Q, Wang J. 2020. Advances in the research on the targets of anti-malaria actions of artemisinin. Pharmacol Ther 216:107697. doi:10.1016/j.pharmthera.2020.107697 33035577 PMC7537645

[67] Bertling K, Banerjee A, Saffarini D. 2021. Aerobic respiration and its regulation in the metal reducer Shewanella oneidensis Front Microbiol 12:723835. doi:10.3389/fmicb.2021.723835 34566926 PMC8458880

[68] Clarke TA, Edwards MJ, Gates AJ, Hall A, White GF, Bradley J, Reardon CL, Shi L, Beliaev AS, Marshall MJ, Wang Z, Watmough NJ, Fredrickson JK, Zachara JM, Butt JN, Richardson DJ. 2011. Structure of a bacterial cell surface decaheme electron conduit. Proc Natl Acad Sci USA 108:9384–9389. doi:10.1073/pnas.1017200108 21606337 PMC3111324

[69] Fredrickson JK, Romine MF. 2005. Genome-assisted analysis of dissimilatory metal-reducing bacteria. Curr Opin Biotechnol 16:269–274. doi:10.1016/j.copbio.2005.04.001 15961027

[70] Le Laz S, Kpebe A, Bauzan M, Lignon S, Rousset M, Brugna M. 2016. Expression of terminal oxidases under nutrient-starved conditions in Shewanella oneidensis: detection of the A-type cytochrome c oxidase. Sci Rep 6:19726. doi:10.1038/srep19726 26815910 PMC4728554

[71] Refojo PN, Sena FV, Calisto F, Sousa FM, Pereira MM. 2019. The plethora of membrane respiratory chains in the phyla of life. Adv Microb Physiol 74:331–414. doi:10.1016/bs.ampbs.2019.03.002 31126533

[72] ALBERT A, MAGRATH D. 1947. The choice of a chelating agent for inactivating trace metals; derivatives of oxine (beta-hydroxyquinoline). Biochem J 41:534–545. 20270788

[73] Chen W-H, Wang M, Yu S-S, Su L, Zhu D-M, She J-Q, Cao X-J, Ruan D-Y. 2007. Clioquinol and vitamin B12 (cobalamin) synergistically rescue the lead-induced impairments of synaptic plasticity in hippocampal dentate gyrus area of the anesthetized rats in vivo. Neuroscience 147:853–864. doi:10.1016/j.neuroscience.2007.04.042 17555879

[74] Tjälve H, Ståhl K. 1984. Effect of 5-chloro-7-iodo-8-hydroxy-quinoline (clioquinol) on the uptake and distribution of nickel, zinc and mercury in mice. Acta Pharmacol Toxicol (Copenh) 55:65–72. doi:10.1111/j.1600-0773.1984.tb01963.x 6235722

[75] Zhu C, Wang Y, Mao Q, Li F, Li Y, Chen C. 2017. Two 8-hydroxyquinolinate based supramolecular coordination compounds: synthesis, structures and spectral properties. Materials (Basel) 10:313. doi:10.3390/ma10030313 28772672 PMC5503320

[76] Babbitt SE, Hsu J, Kranz RG. 2016. Molecular basis behind inability of mitochondrial holocytochrome c synthase to mature bacterial cytochromes: defining a critical role for cytochrome c α helix-1. J Biol Chem 291:17523–17534. doi:10.1074/jbc.M116.741231 27387500 PMC5016149

[77] Verissimo AF, Sanders J, Daldal F, Sanders C. 2012. Engineering a prokaryotic apocytochrome c as an efficient substrate for Saccharomyces cerevisiae cytochrome c heme lyase. Biochem Biophys Res Commun 424:130–135. doi:10.1016/j.bbrc.2012.06.088 22732413 PMC4420188

[78] Laleve A, Panozzo C, Kühl I, Bourand-Plantefol A, Ostojic J, Sissoko A, Tribouillard-Tanvier D, Cornu D, Burg A, Meunier B, Blondel M, Clain J, Bonnefoy N, Duval R, Dujardin G. 2020. Artemisinin and its derivatives target mitochondrial c-type cytochromes in yeast and human cells. Biochim Biophys Acta Mol Cell Res 1867:118661. doi:10.1016/j.bbamcr.2020.118661 31987792

[79] Chung I-Y, Jang H-J, Yoo Y-J, Hur J, Oh H-Y, Kim S-H, Cho Y-H. 2022. Artemisinin displays bactericidal activity via copper-mediated DNA damage. Virulence 13:149–159. doi:10.1080/21505594.2021.2021643 34983312 PMC8741286

[80] Bareggi SR, Cornelli U. 2012. Clioquinol: review of its mechanisms of action and clinical uses in neurodegenerative disorders. CNS Neurosci Therapeut 18:41–46. doi:10.1111/j.1755-5949.2010.00231.x PMC6493473 21199452

[81] Wykowski R, Fuentefria AM, de Andrade SF. 2022. Antimicrobial activity of clioquinol and nitroxoline: a scoping review. Arch Microbiol 204:535. doi:10.1007/s00203-022-03122-2 35907036 PMC9362210

[82] Huang X, Li Q, Yun S, Guo J, Yang H, Wang J, Cheng J, Sun Z. 2024. Zn(II) enhances the antimicrobial effect of chloroxine and structural analogues against drug-resistant ESKAPE pathogens in vitro. Biochem Pharmacol 229:116482. doi:10.1016/j.bcp.2024.116482 39134284

[83] Borisov VB, Giardina G, Pistoia G, Forte E. 2025. Cytochrome bd-type oxidases and environmental stressors in microbial physiology. Adv Microb Physiol 86:199–255. doi:10.1016/bs.ampbs.2024.05.001 40404270

[84] Burton JAJ, Edwards MJ, Richardson DJ, Clarke TA. 2025. Electron transport across bacterial cell envelopes. Annu Rev Biochem 94:89–109. doi:10.1146/annurev-biochem-052621-092202 40096216

[85] Lahiri R, Adams LB, Thomas SS, Pethe K. 2022. Sensitivity of Mycobacterium leprae to Telacebec. Emerg Infect Dis 28:749–751. doi:10.3201/eid2803.210394 35202539 PMC8888226

[86] Lee Bei Shi, Hards K, Engelhart CA, Hasenoehrl EJ, Kalia NP, Mackenzie JS, Sviriaeva E, Chong SMS, Manimekalai MSS, Koh VH, Chan J, Xu J, Alonso S, Miller MJ, Steyn AJC, Grüber G, Schnappinger D, Berney M, Cook GM, Moraski GC, Pethe K. 2021. Dual inhibition of the terminal oxidases eradicates antibiotic-tolerant Mycobacterium tuberculosis. EMBO Mol Med 13:e13207. doi:10.15252/emmm.202013207 33283973 PMC7799364

[87] Lee B.S, Sviriaeva E, Pethe K. 2020. Targeting the cytochrome oxidases for drug development in mycobacteria. Prog Biophys Mol Biol 152:45–54. doi:10.1016/j.pbiomolbio.2020.02.001 32081616

[88] Harrison GA, Mayer Bridwell AE, Singh M, Jayaraman K, Weiss LA, Kinsella RL, Aneke JS, Flentie K, Schene ME, Gaggioli M, Solomon SD, Wildman SA, Meyers MJ, Stallings CL. 2019. Identification of 4-amino-thieno[2,3-d]pyrimidines as QcrB inhibitors in Mycobacterium tuberculosis. mSphere 4:e00606-19. doi:10.1128/mSphere.00606-19 31511370 PMC6739496

[89] Grauel A, Kägi J, Rasmussen T, Makarchuk I, Oppermann S, Moumbock AFA, Wohlwend D, Müller R, Melin F, Günther S, Hellwig P, Böttcher B, Friedrich T. 2021. Structure of Escherichia coli cytochrome bd-II type oxidase with bound aurachin D. Nat Commun 12:6498. doi:10.1038/s41467-021-26835-2 34764272 PMC8585947

[90] Jeffreys LN, Ardrey A, Hafiz TA, Dyer L-A, Warman AJ, Mosallam N, Nixon GL, Fisher NE, Hong WD, Leung SC, Aljayyoussi G, Bibby J, Almeida DV, Converse PJ, Fotouhi N, Berry NG, Nuermberger EL, Upton AM, O’Neill PM, Ward SA, Biagini GA. 2023. Identification of 2-aryl-quinolone inhibitors of cytochrome bd and chemical validation of combination strategies for respiratory inhibitors against Mycobacterium tuberculosis ACS Infect Dis 9:221–238. doi:10.1021/acsinfecdis.2c00283 36606559 PMC9926492

[91] Seitz C, Ahn S-H, Wei H, Kyte M, Cook GM, Krause KL, McCammon JA. 2024. Targeting tuberculosis: novel scaffolds for inhibiting cytochrome bd oxidase. J Chem Inf Model 64:5232–5241. doi:10.1021/acs.jcim.4c00344 38874541

[92] Jackson LK, Dailey TA, Anderle B, Warren MJ, Bergonia HA, Dailey HA, Phillips JD. 2023. Exploiting differences in heme biosynthesis between bacterial species to screen for novel antimicrobials. Biomolecules 13:1485. doi:10.3390/biom13101485 37892169 PMC10604556

[93] Schneider CA, Rasband WS, Eliceiri KW. 2012. NIH Image to ImageJ: 25 years of image analysis. Nat Methods 9:671–675. doi:10.1038/nmeth.2089 22930834 PMC5554542

[94] Berida T, McKee SR, Chatterjee S, Manning DL, Li W, Pandey P, Tripathi SK, Mreyoud Y, Smirnov A, Doerksen RJ, Jackson M, Ducho C, Stallings CL, Roy S. 2023. Discovery, synthesis, and optimization of 1,2,4-triazolyl pyridines targeting Mycobacterium tuberculosis. ACS Infect Dis 9:2282–2298. doi:10.1021/acsinfecdis.3c00341 37788674 PMC10807233

[95] Sarkar S, Mayer Bridwell AE, Good JAD, Wang ER, McKee SR, Valenta J, Harrison GA, Flentie KN, Henry FL, Wixe T, Demirel P, Vagolu SK, Chatagnon J, Machelart A, Brodin P, Tønjum T, Stallings CL, Almqvist F. 2023. Design, synthesis, and evaluation of novel Δ^2^-thiazolino 2-pyridone derivatives that potentiate isoniazid activity in an isoniazid-resistant Mycobacterium tuberculosis mutant. J Med Chem 66:11056–11077. doi:10.1021/acs.jmedchem.3c00358 37485869 PMC10461229

