# Supplementary material for: Inhibitors of cytochrome c biogenesis pathways
Source: mBio. 2026 Apr 20;17(5):e00273-26. doi: 10.1128/mbio.00273-26 (PMC13170271; doi:10.1128/mbio.00273-26)

Supplemental Material

Title: Inhibitors of cytochrome c biogenesis pathways

Authors: Deanna L. Mendez[1], Pema L. Childs[1], Amidala J. Martinie[1], Jonathan Q. Huynh[1], Andy F. Zhu[1], Samuel R. McKee[2], George S. Ghabrial[2], Christina L. Stallings[2], Robert G. Kranz[1]

1. Department of Biology, Washington University in St. Louis, St. Louis, MO 63130, USA

2. Department of Molecular Microbiology, Washington University School of Medicine, Saint Louis, MO 63110 USA

2. Center for Women's Infectious Disease Research, Washington University School of Medicine, Saint Louis, MO 63110 USA

#address correspondence to

Robert G. Kranz

Campus Box 1137

One Brookings Drive

St. Louis, MO 63130

Phone: +1 (314) 935-4278

Email: kranz@wustl.edu

Table S1. Molecular structures of candidate drugs shown in Table 1.

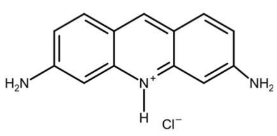<br>A. Acriflaviunium HCl	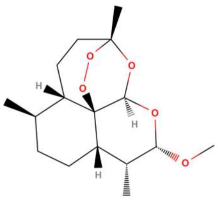<br>B. Artemether	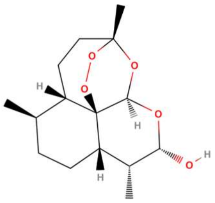<br>C. Artenimol	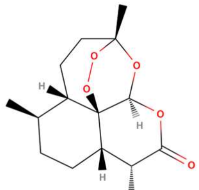<br>D. Artemisinin	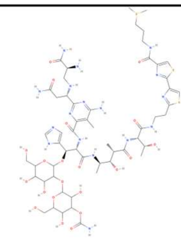<br>E. Bleomycin
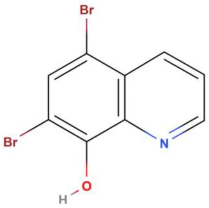<br>F. Broxyquinoline	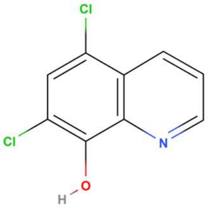<br>G. Chloroxine	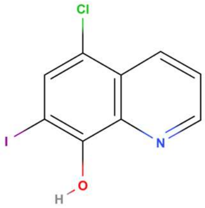<br>H. Clioquinol	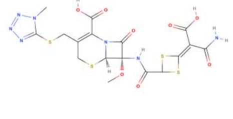<br>I. Cefotetan	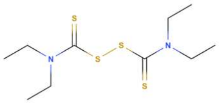<br>J. Disulfiram
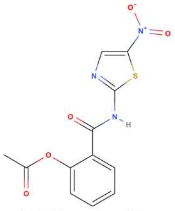<br>K. Nitazoxanide	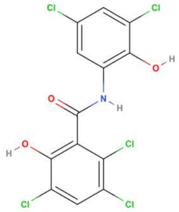<br>L. Oxyclozanide	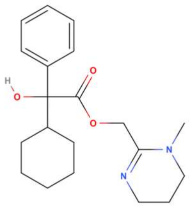<br>M. Oxyphencyclimine	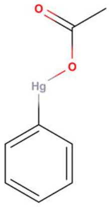<br>N. Phenylmercuric Acetate	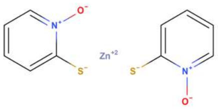<br>O. Pyrithione Zinc

Figure S1. Screen of the Pharmakon 1760 library for inhibitors of Systems I and II.

A. Timing, growth, and assay to detect inhibitors of cyt c maturation. B. *E. coli* strains for assays used in the Pharmakon 1760 screen and subsequent follow-up experiments. The endogenous, natural ccm genes have been deleted to investigate the recombinant expression of System I, II, or III. The System proteins (CcsBA, CcmA-H, and HCCS in pGEX) are inducible with IPTG, and the cyt c_4 (cyt c for Sys III) reporter is inducible with arabinose. C. Heme stains of a subset of the screen. Candidate drugs from Table 1 are highlighted in blue and red here. Rifaximin is colored green, a known transcriptional inhibitor.

A

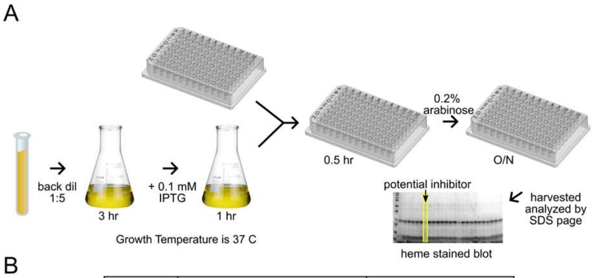

B

Strain		Reference
C43(DE3)	F-ompT hsdSB (rB- mB-) gal dcm (DE3)	Miroux and Walker, 1996
MS404	C43 Δccm::KanR+ pGEX vector::CarbR+ pBAD-cyt c4-His::CmR	Feissner et al, 2006
MS111	C43 Δccm::KanR+ pGEX-GST*CcsBA-His::CarbR+ pBAD-cyt c4-His::CmR	Sutherland et al, 2021
MS146	C43 Δccm::KanR+ pGEX-GST-SysI(ccmABCDEFGH)::CarbR+ pBAD-cyt c4-His::CmR	Feissner et al, 2006
MS079	MG1655 Δccm::KanR+ pGEX-GST-HCCS::CarbR+pBAD-CYCS::CmR	San Francisco et al, 2012

C

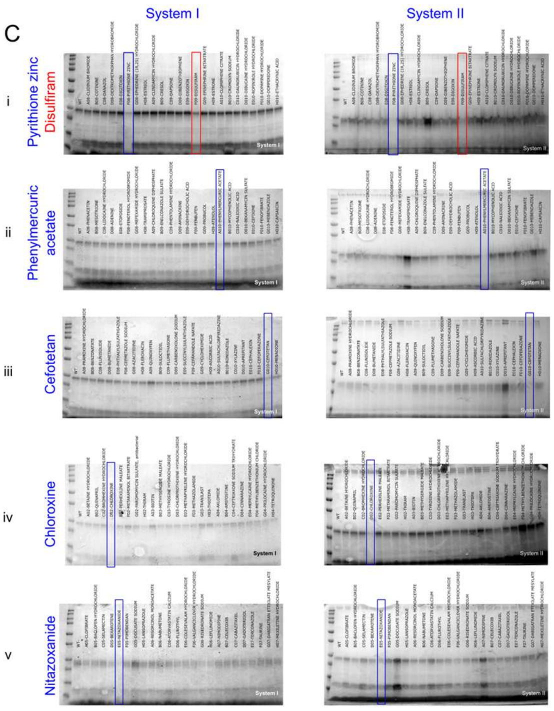

C

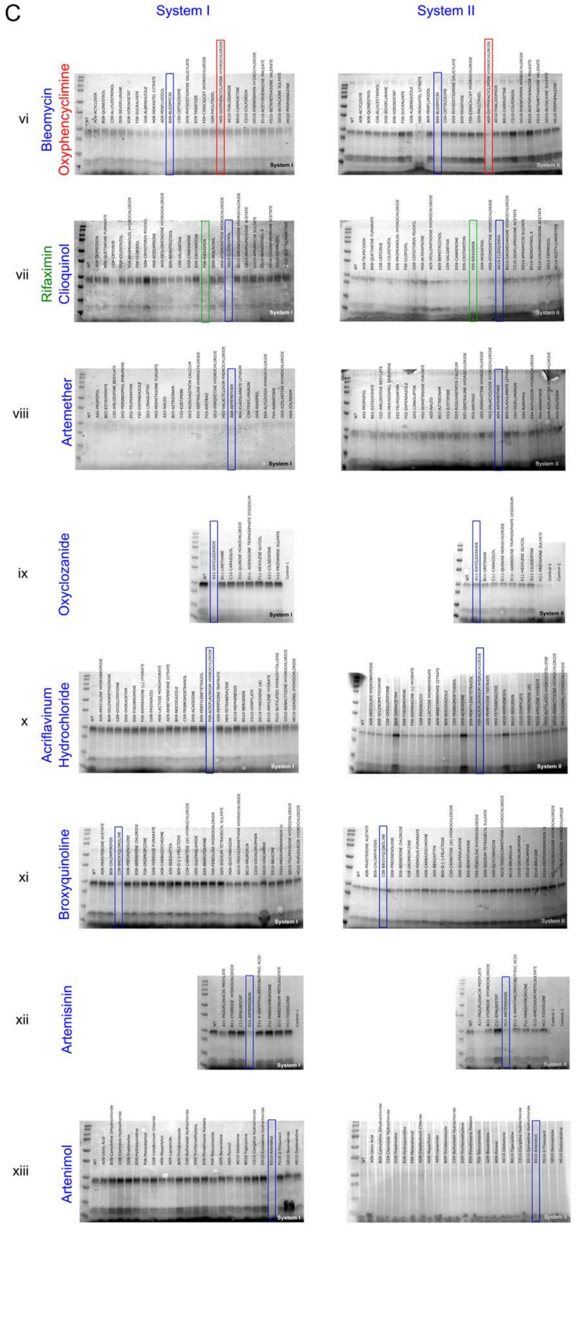

Figure S2: Spectroscopic signatures of heme from in vitro assays from Sys II and III in the presence of candidate drugs.

A. Apocyt c was matured in the presence of Pyrithione Zinc (ZnP 7.5, 75μM), Disulfiram (67μM), but not Artemether or Artemisinin. Panels Av-vii are representative of three independent experiments. B. Apocytc 56-mer was matured in the presence of Chloroxine (67μM), Clioquinol (67μM), but not in the presence of Artemether (67μM) or Artemisinin (67μM). C,D. The maturation of Apocytc 56-mer by CcsBA or HCCS was inversely related to the amount of Artemether/Artemisinin present. Panels C vii and D vii show mean values from two independent experiments. The error bars represent the standard deviation. The corresponding heme stains are found in Supp. Fig. 3

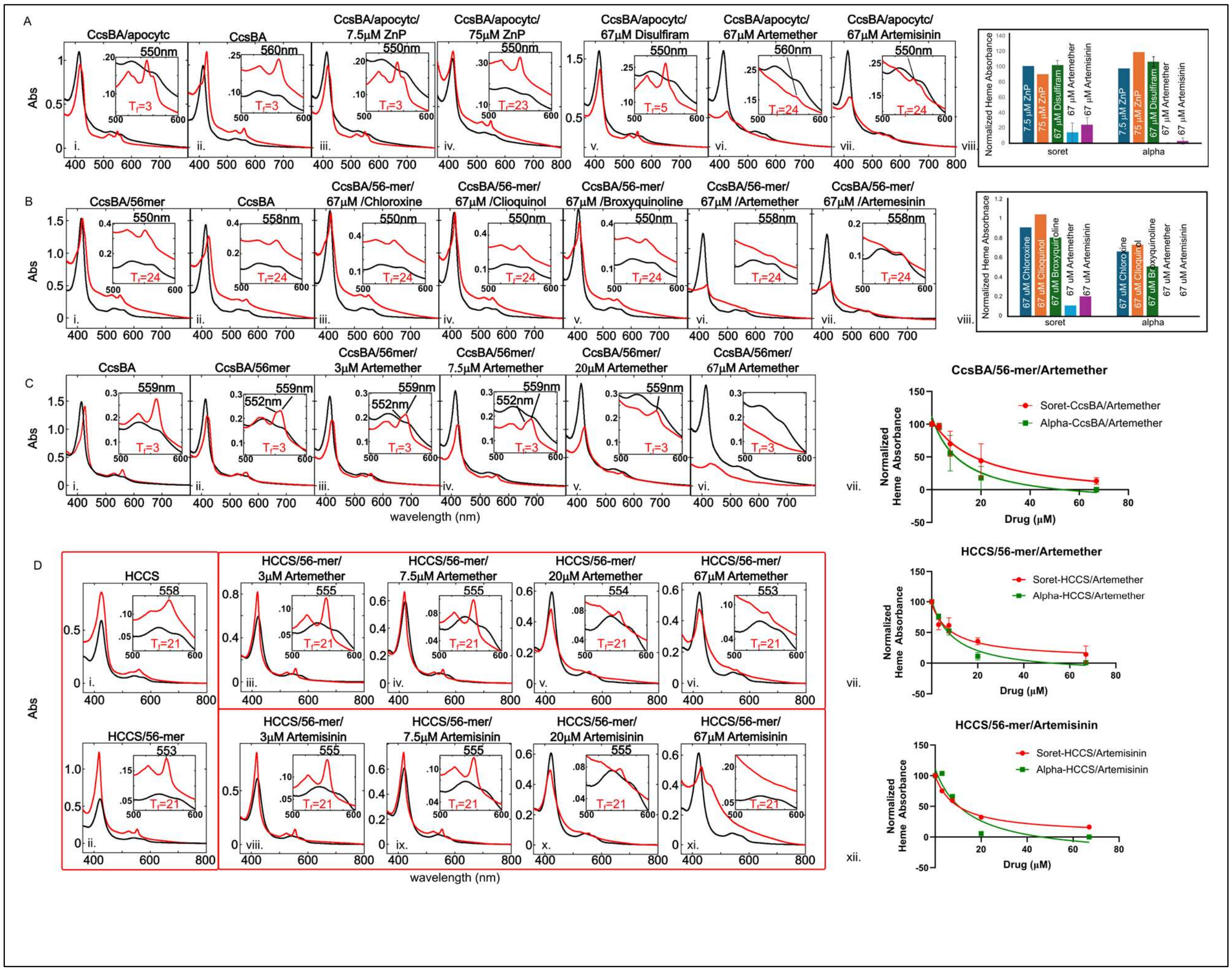

Figure S3: ***In vitro*** **cyt c biogenesis as monitored by heme stain in the presence of candidate drugs.**

Panels A-D. are the complete panels corresponding to the data in Figure 3. Heme stains are false colored red while the Coomassie stained gel was false colored blue. On a technical note, the Coomassie was performed on the gel after the heme stain and in some cases the heme stain is visible on the Coomassie stained gel. The bands corresponding to the cyt c biogenesis system are labeled on the sides of the Coomassie gel as are the heme stainable protein/peptide. Unlabeled staining at the bottom of the gel corresponds to free heme.

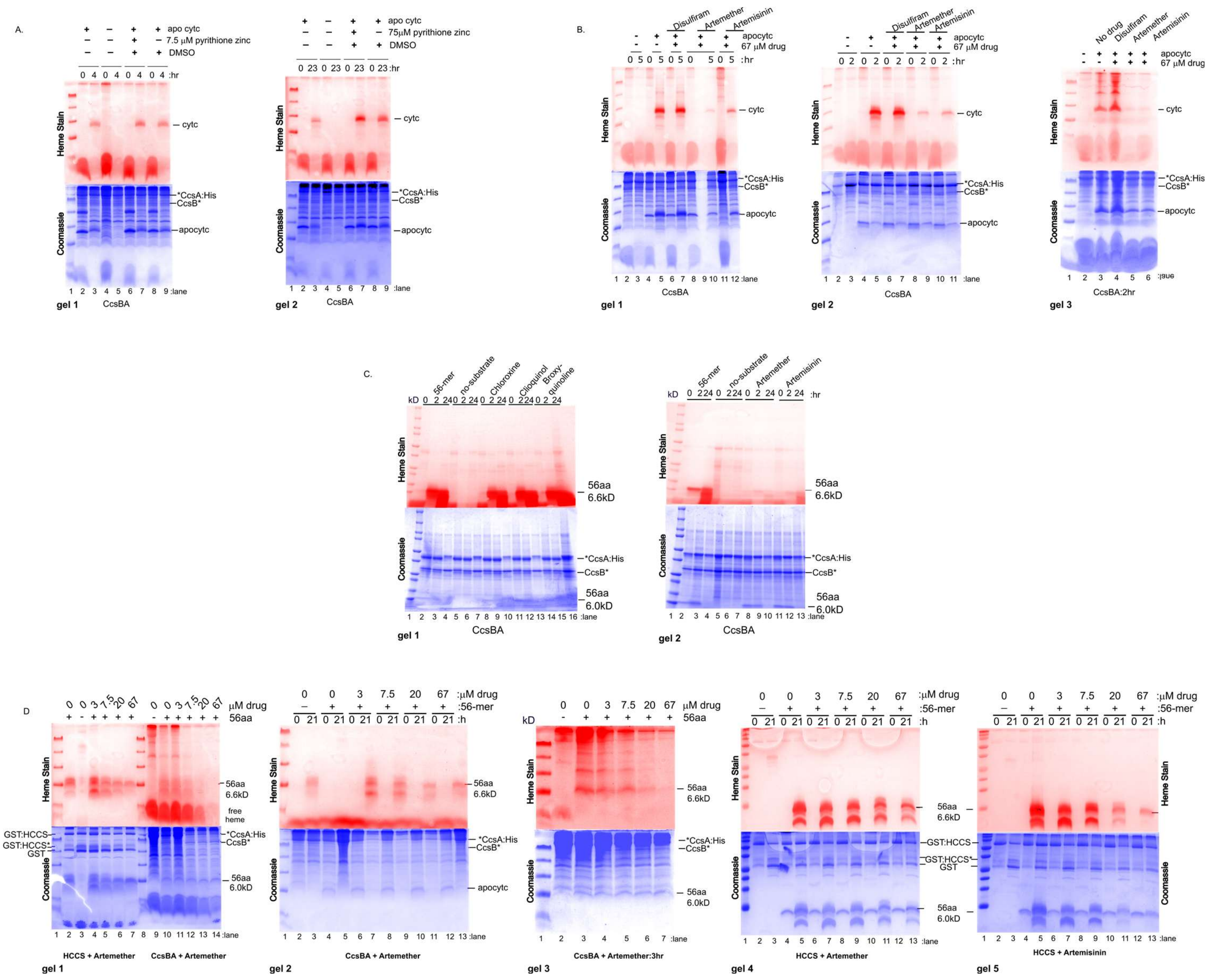

Figure S4. Plots of the normalized bacterial culture OD 600nm as a function of drug concentration.

Recombinant *E. coli* with the indicated System were grown in 6ml cultures and drug was added. To measure total cell density, OD600 prior to drug addition and once the experiment was completed was measured. The normalized OD 600 was calculated by the following equation: (OD600 final/ OD600 initial)/(no drug OD600 final/ no drug OD600 initial). The error bars are the standard deviation from three independent trials.

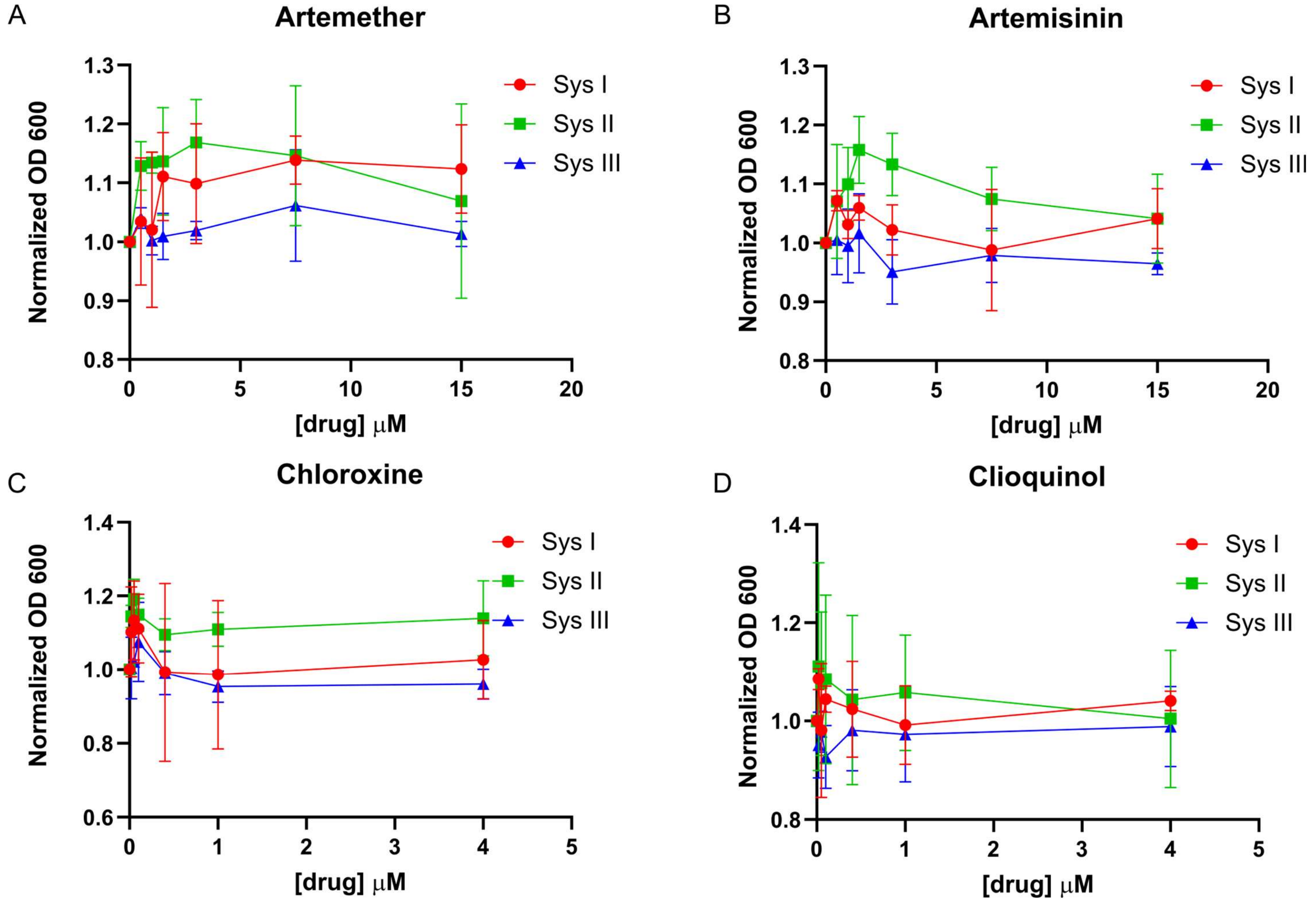

Figure S5. Heme stain blots used to calculate IC_{50}s for cyt c biogenesis pathways. Recombinant *E.coli* with each system were challenged with the indicated drug, followed by cyt c assays, as described in the text. Results of assays are compiled in Fig 4A and B.

Below each blot are the boxes and heme stain intensities determined by Licor Image Studio.

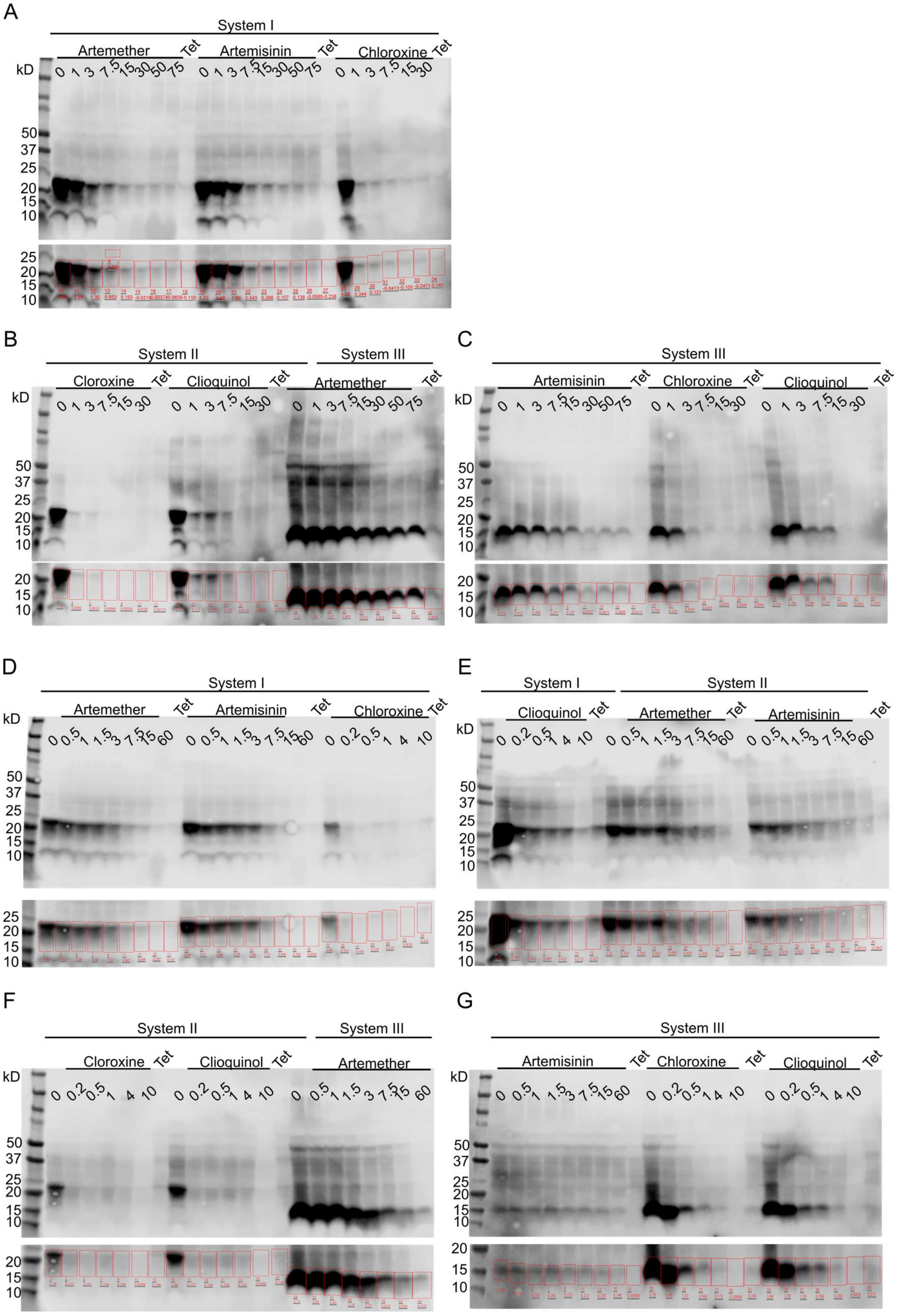

Figure S5. continued

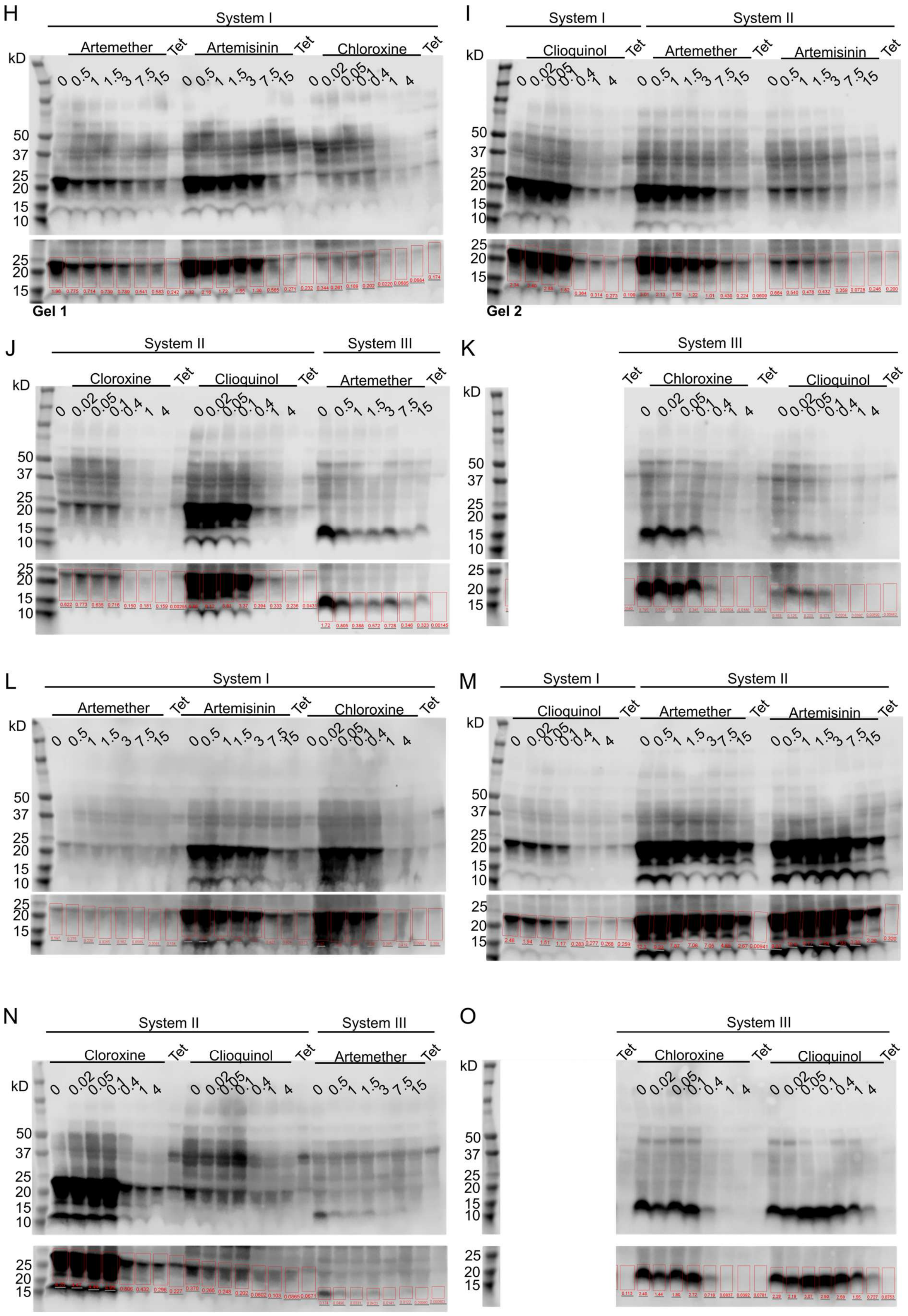

Figure S5. continued

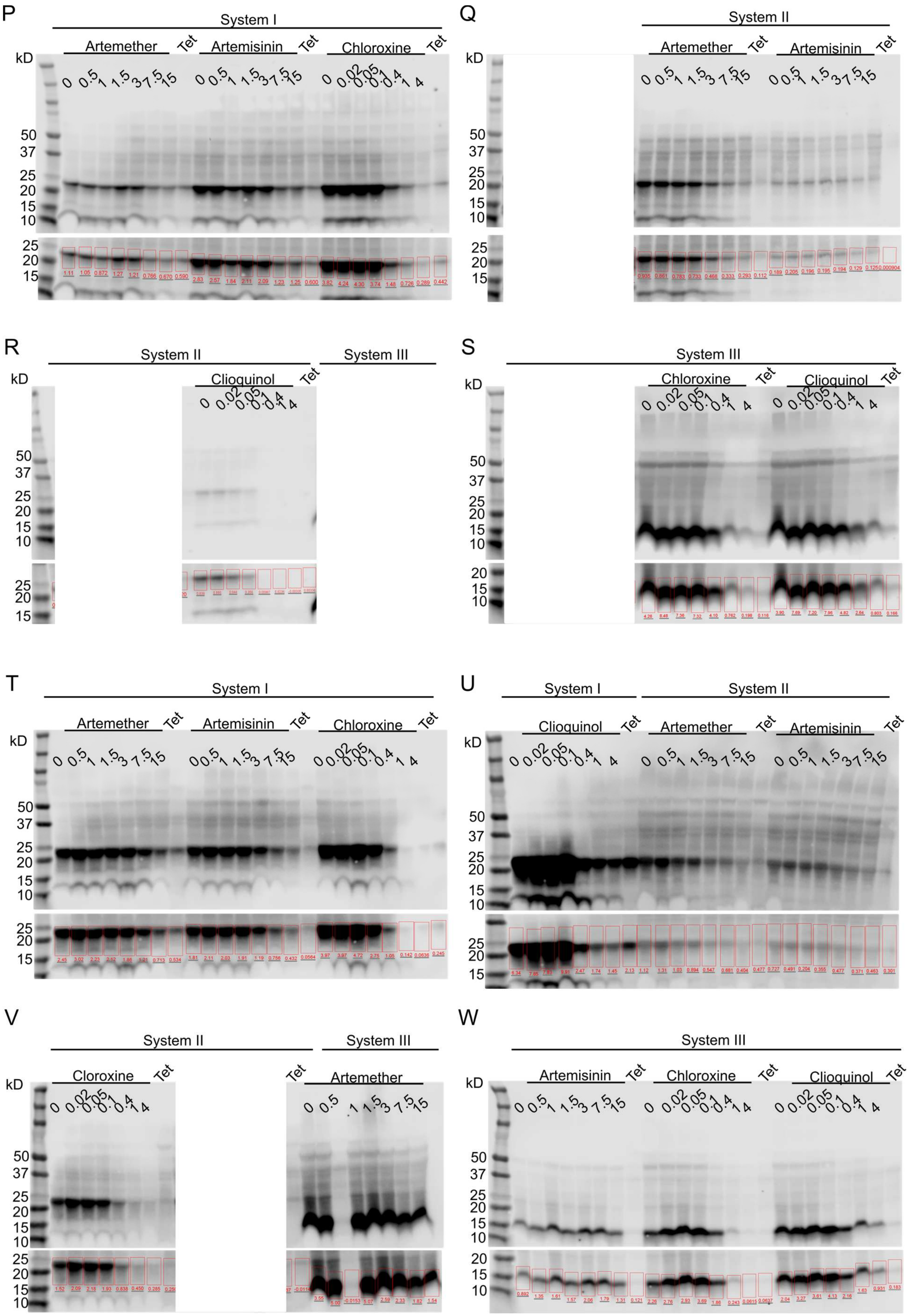

Figure S5. continued

X

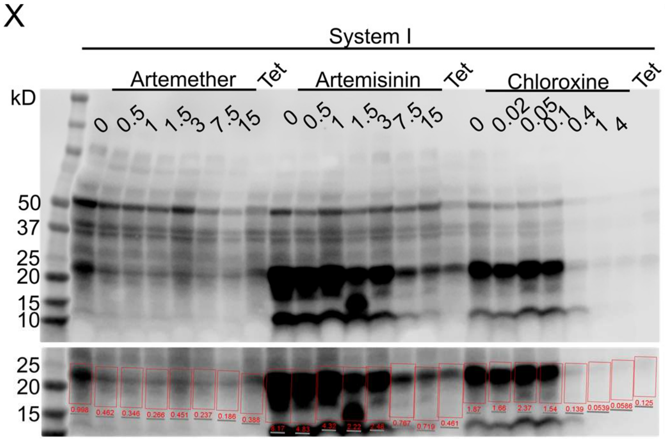

Y

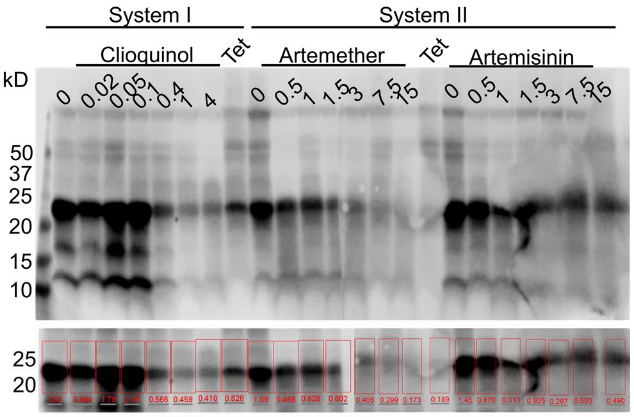

Z

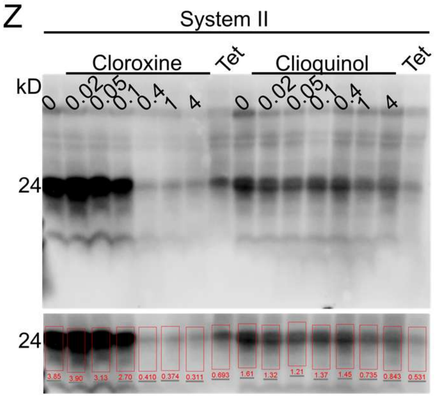

AA

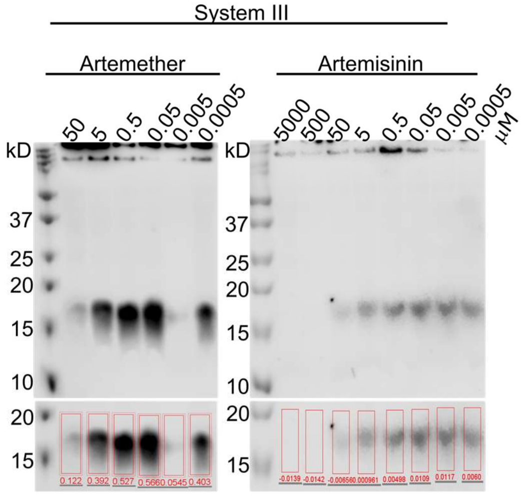

BB

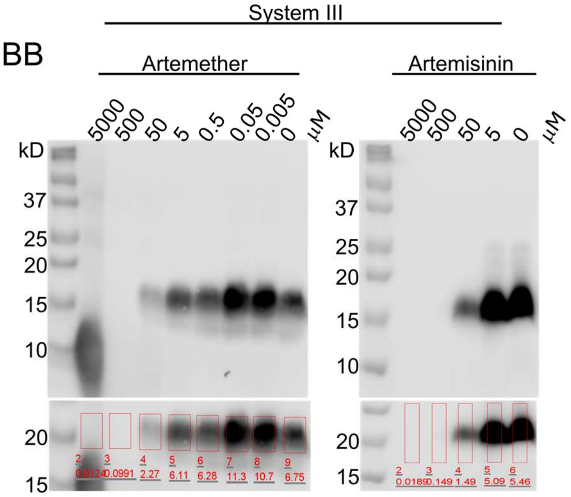

Figure S6. Images of plates used to measure the zones of inhibition shown in Figure 5.

The numbers on the plates correspond to the following drugs, 1-Artemether, 2-Artemisinin, 3-Disulfiram, 4-Pyrithione Zinc (ZnP), 5-Phenyl Mercuric Acetate (PMA), 6-Chloroxine, 7- Clioquinol, 8-Broxyquinoline. The dose is provided on the Figure 5 Table headings.

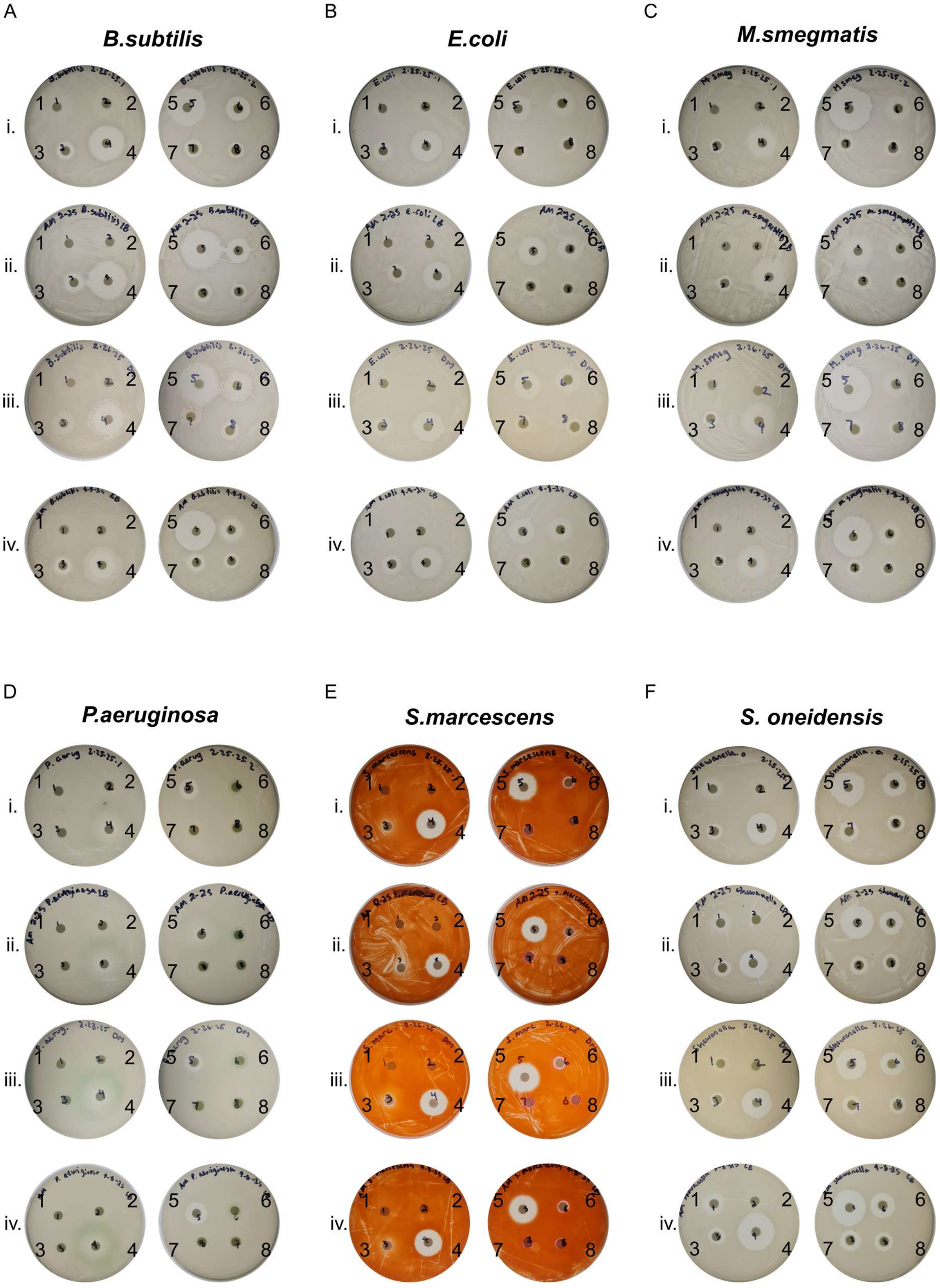

Figure S7. Quinolines and chelators exhibit different trends in their zones of inhibition across bacteria.

Zone of inhibition experiments were performed with six different bacteria and response to the chelators shown below were recorded. Mean, standard deviation (SD) and the number of replicates (N) are shown in the table and plotted.

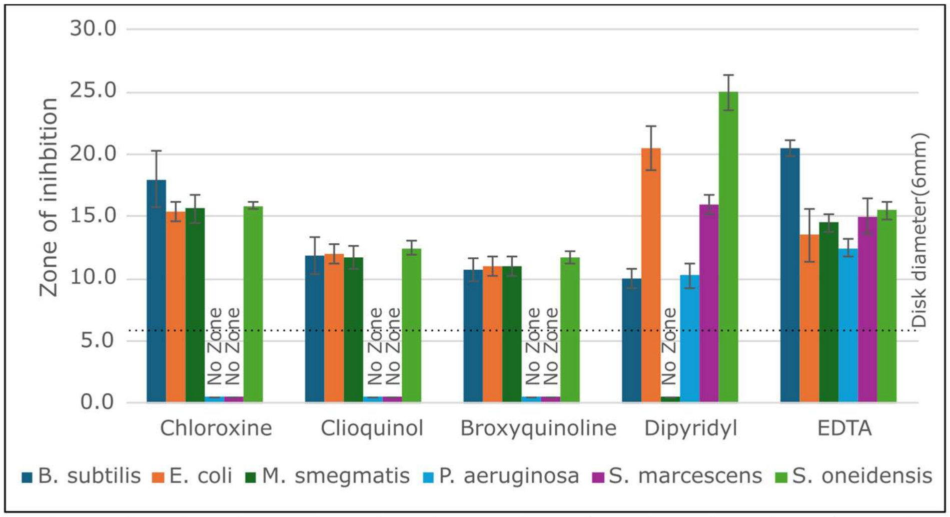

	Chloroxine 100nmol			Clioquinol 200nmol			Broxyquinoline 200nmol			Dipyridyl 100nmol			EDTA 625nmol		
	**Mean**	**SD**	**N**	**Mean**	**SD**	**N**	**Mean**	**SD**	**N**	**Mean**	**SD**	**N**	**Mean**	**SD**	**N**
B. subtilis	18.0	2.3	4.0	11.9	1.4	4.0	10.8	1.0	4.0	10.0	0.8	4.0	20.5	0.7	2.0
E. coli	15.4	0.8	4.0	12.0	0.8	4.0	11.0	0.8	4.0	20.5	1.7	4.0	13.5	2.1	2.0
M. smegmatis	15.6	1.1	4.0	11.8	1.0	4.0	11.0	0.8	4.0	0.0	0.0	4.0	14.5	0.7	2.0
P. aeruginosa	0.0	0.0	4.0	0.0	0.0	4.0	0.0	0.0	4.0	10.3	1.0	4.0	12.5	0.7	2.0
S. marcescens	0.0	0.0	4.0	0.0	0.0	4.0	0.0	0.0	4.0	16.0	0.8	4.0	15.0	1.4	2.0
S. oneidensis	15.9	0.3	4.0	12.5	0.6	4.0	11.8	0.5	4.0	25.0	1.4	2.0	15.5	0.7	2.0

Figure S8. Clioquinol and Chloroxine demonstrate greater potency against *M. tuberculosis* than Artemether or Artemisinin.

Wild-type *M. tuberculosis* Erdman was incubated with serial dilutions of each compound for 7 days, and bacterial respiration was assessed using the Microplate Alamar Blue Assay (MABA). Data represent mean values ± SD from four biological replicates (n = 4).

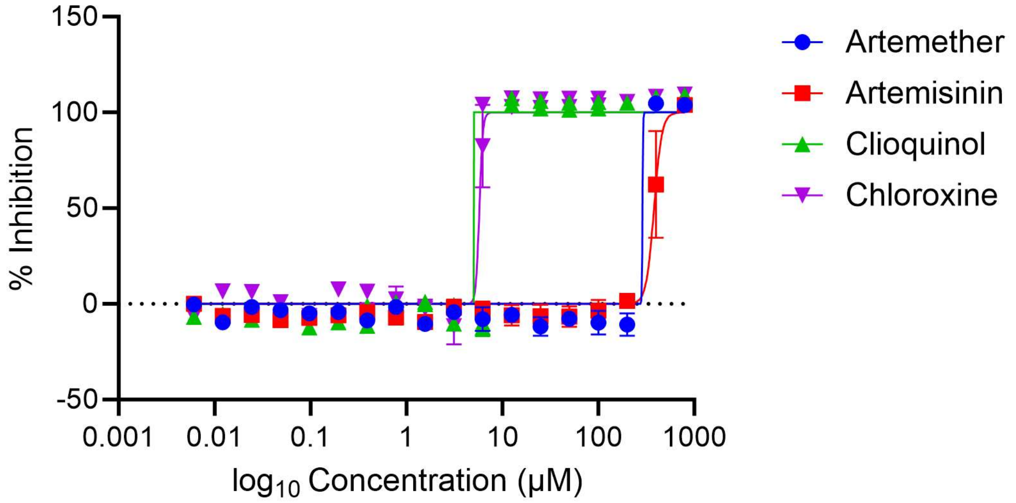

Figure S9. Artemether inhibits the biosysnthesis of c-type cytochromes *in vivo* in *Shewanella oneidensis*.

A. Heme and Sypro stain of total cell lysate run on SDS-page separating gel. SuperSignal™ West Fempto substrate was used to detect the presence of heme stainable proteins in *S. oneidensis*. **B**. The UV-vis absorbance spectra of the samples in "A" showing that heme (from cyt c) decreases with greater Artemether concentrations. **C**. The 95% confidence interval for the IC_{50} value calculated in Prism with nonlinear regression based on Heme stain, soret absorbance, and alpha peak. **A, B** are representative of three independent trials.

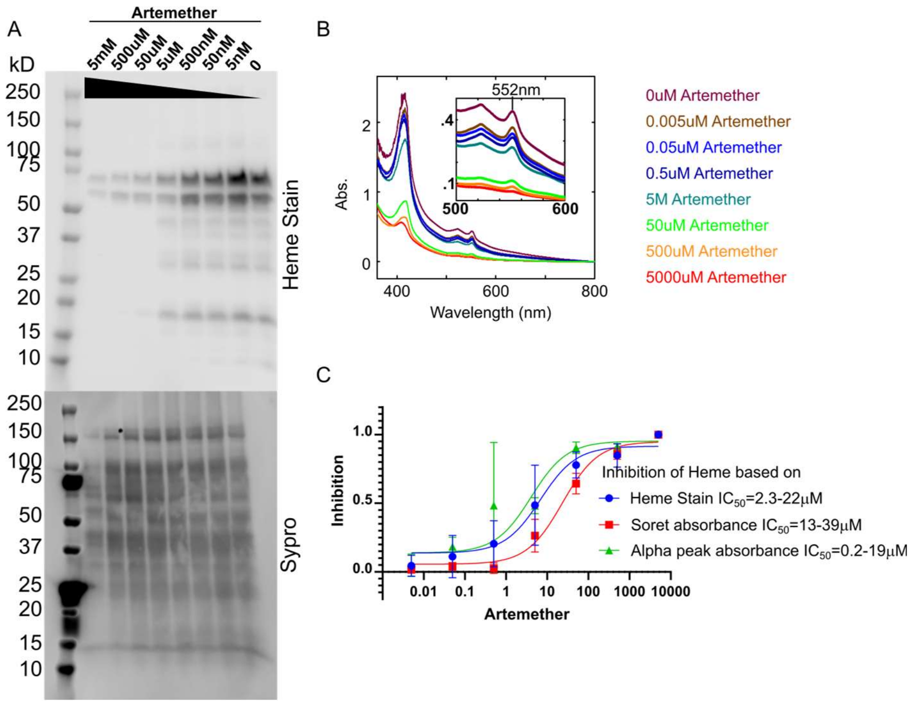

Supplement: Supplemental material — Table S1; Figures S1-S9. [file mbio.00273-26-s0001.pdf]
